# Externally validated clinical prediction models for estimating treatment outcomes for patients with a mood, anxiety or psychotic disorder: systematic review and meta-analysis

**DOI:** 10.1192/bjo.2024.789

**Published:** 2024-12-05

**Authors:** Desi G. Burghoorn, Sanne H. Booij, Robert A. Schoevers, Harriëtte Riese

**Affiliations:** University Medical Center Groningen, Department of Psychiatry, Interdisciplinary Center Psychopathology and Emotion Regulation (ICPE), University of Groningen, Groningen, The Netherlands

**Keywords:** Anxiety or fear-related disorders, depressive disorders, psychotic disorders/schizophrenia, systematic review, meta-analysis

## Abstract

**Background:**

Suboptimal treatment outcomes contribute to the high disease burden of mood, anxiety or psychotic disorders. Clinical prediction models could optimise treatment allocation, which may result in better outcomes. Whereas ample research on prediction models is performed, model performance in other clinical contexts (i.e. external validation) is rarely examined. This gap hampers generalisability and as such implementation in clinical practice.

**Aims:**

Systematically appraise studies on externally validated clinical prediction models for estimated treatment outcomes for mood, anxiety and psychotic disorders by (1) reviewing methodological quality and applicability of studies and (2) investigating how model properties relate to differences in model performance.

**Method:**

The review and meta-analysis protocol was prospectively registered with PROSPERO (registration number CRD42022307987). A search was conducted on 8 November 2021 in the databases PubMED, PsycINFO and EMBASE. Random-effects meta-analysis and meta-regression were conducted to examine between-study heterogeneity in discriminative performance and its relevant influencing factors.

**Results:**

Twenty-eight studies were included. The majority of studies (*n* = 16) validated models for mood disorders. Clinical predictors (e.g. symptom severity) were most frequently included (*n* = 25). Low methodological and applicability concerns were found for two studies. The overall discrimination performance of the meta-analysis was fair with wide prediction intervals (0.72 [0.46; 0.89]). The between-study heterogeneity was not explained by number or type of predictors but by disorder diagnosis.

**Conclusions:**

Few models seem ready for further implementation in clinical practice to aid treatment allocation. Besides the need for more external validation studies, we recommend close examination of the clinical setting before model implementation.

For almost three decades, anxiety, mood and psychotic disorders formed over 14% of the age-standardised years lived with disabilities (YLDs) globally.^1^ The high burden of these disorders is, beyond the high prevalence, because of the high number of non-responding patients to initial or subsequent treatments.^2^ Most patients do not remit after their first treatment, and typically multiple therapies have failed before finding one that works.^2^ For patients, this means enduring a longer period of ineffectively treated symptoms and discomfort. For society, prolonged treatment duration puts a strain on the limited resources of mental healthcare services, causing long waiting lists for those who seek care.

## Precision psychiatry

Prolonged treatment duration is related to the low clinical utility of available diagnostic systems to guide treatment choices.^2^ Classification systems such as the ICD-10 and DSM are descriptive in nature, based on experienced symptoms.^3,4^ As such, individuals with the same diagnosis receiving similar treatments may not necessarily share biopsychosocial psychopathological mechanisms. Consequently, treatment responses vary widely.^2,4^ To surpass this heterogeneity, the promise of precision medicine into psychiatry, defined as ‘to integrate clinical data with patient characteristics to uncover disease subtypes and improve the accuracy with which patients are categorized and treated’, is very appealing.^5^ By integrating clinical data with relevant biological, psychological or social patient information, more specific biopsychosocial markers can be established for better treatment response prediction and, consequently, improvement of outcomes.^6,7^

## Clinical prediction models

Over the past two decades, precision psychiatry has gained momentum and, as such, a wealth of clinical prediction models for personalised treatment outcomes have been developed.^8^ However, there is a large translational gap between model development and implementation in clinical practice, preventing successful implementation.

## Requirements for successful prediction model implementation

For a prediction model to have added value in clinical practice, the model first needs to have adequate discriminative ability^a^ and calibration^b^.^9^ Second, a model should be generalisable to different clinical contexts by external validation (e.g. location, treatment setting or period).^9^ Third, successful implementation depends on applicability factors, such as the feasibility to obtain relevant predictors within a clinical context in which time and financial constraints are common.^10^ Previous systematic reviews into clinical prediction models in psychiatry shed light on the above-mentioned factors; either for a broad class of prediction models in psychiatry, including diagnostic models and models predicting onset, or for treatment outcome prediction in depression only.^8,11^ In addition, all focused on a broad range of studies (e.g. from predictor-finding or development to external validation or implementation), and only one review assessed heterogeneity between studies in a quantitative way for a broad range of studies.

## Aims

This important knowledge gap regarding the prediction accuracy, methodological quality, applicability and potential sources of heterogeneity for externally validated models for treatment outcomes of common mental disorders is addressed in the current systematic review and meta-regression. We will focus on three aims. First, we systematically appraise the current literature on externally validated clinical prediction models that estimate treatment outcomes for mood, anxiety and psychotic disorders, to qualitatively describe variability across studies and their models. This includes, among other study properties, a systematic categorisation of included predictor types, as precision psychiatry aims to develop prediction models containing relevant predictors, which, according to the biopsychosocial model of mental disorders,^12^ should be biological and psychosocial, next to clinical predictors. Second, we critically review the methodological quality and applicability of included studies using the Prediction Model Risk of Bias Assessment Tool (PROBAST), to ensure proper interpretation of study results and to determine the validity and applicability of prediction models. Third, given the broad inclusion criteria of the included studies, data are expected to be heterogeneous on characteristics such as patients’ disorders, predictors and study settings.^13^ Therefore, a meta-analysis to estimate the average discriminative ability is not deemed useful.^13^ However, the observed heterogeneity itself and its sources are informative for investigating how clinical and methodological aspects of the studies relate to their results.^14^ For this reason, we conduct a meta-analysis on the omnibus discrimination performance to estimate how well the currently available models perform overall, concurrently focusing on the observed heterogeneity. This is informative for investigating how clinical and methodological aspects of the studies relate to their results.^14^ Hence, if heterogeneity is confirmed, the fourth aim is to examine whether heterogeneity in discrimination performance can be explained by a meta-regression on various study characteristics.

## Method

The systematic review and meta-analysis were conducted conform the e-Cochrane Handbook for Systematic Review of Interventions and the preferred reporting items for systematic reviews^15^ and meta-analysis (PRISMA) statement^16^ (see the research checklist). The protocol was registered in the International Prospective Register of Systematic Reviews (PROSPERO) CRD42022307987 (307987). See Supplementary file 2 available at https://doi.org/10.1192/bjo.2024.789, which describes the scope of this review in terms of patient population, intervention (treatment), comparison, outcome and type of study (PICOT).

### Definitions

The following key concepts are described for a clear and precise understanding of the terminology central to our study.

- Clinical prediction model: a model that is developed to facilitate the prognostic ability estimations in daily medical practice, by (statistically) associating multiple predictors with outcome data from a sample.^9^
- Multivariate model: a model estimating that a specific event (e.g. remission) will occur, based on multiple characteristics or pieces of information for a specific individual.^17^
- Treatment outcome: any type of outcome, whether it be of clinical, psychosocial or biological nature, that could be associated with an effect (or absence thereof) induced by pharmacological or/and psychoeducational treatment.^9^
- External validation: the prediction model is applied to a similar (but not necessarily the same) target population as in the development data-set, by one or more of the following validation methods to ensure the test data-set is independent:^9^
  - temporal: model tested in the same data collection, but using different individuals at a later timepoint;
  - geographical: model is tested in a sample from a different location;
  - different settings: model is tested in a different setting (e.g. from secondary to primary care setting).
- Statistical learning: statistical learning includes the Cox hazard model, logistic regression model, linear regression model, negative binomial model, generalised linear model, Weibull regression model, regularised regression model and other regression methods, either standard, penalised, boosted or bagged.^11^
- Machine learning: machine learning includes classification trees, random forests, artificial neural networks, support vector machines, boosted tree methods, Bayes machine learning algorithms, *K*-nearest neighbours algorithms, multivariate adaptive regression splines and genetic algorithms.^11^

### In- and exclusion criteria

The following inclusion criteria were adopted: (1) studies including adults with a mood, anxiety or psychotic diagnosis as confirmed by diagnostic interview or patient file; (2) studies externally validating clinical prediction models for any type of treatment outcome; and (3) studies testing multivariable models following the transparent reporting of a multivariable prediction model for individual prognosis or diagnosis (TRIPOD) statement.^17^

The following exclusion criteria were adopted: (1) publication types not based on original data (abstracts, conference proceeding, reviews, meta-analyses); (2) predictor-finding studies that did not report prediction models; (3) studies with predictive models that did not evaluate a model's performance for personalised risk prediction in any recommended way;^c^ (4) studies predicting the first onset of disorders; (5) studies including children (under the age of 12) or exclusively focused on adolescents (aged 12–18); (6) studies solely including participants recruited from the general population (i.e. not in mental healthcare settings); (7) studies exclusively focusing on postnatal mental disorders; and (8) studies not written in the English, German or Dutch language. No restrictions were placed based on the model's performance in the development data-set.

### Development of the search string

To ensure conceptual validity, *a priori* 13 must-find PubMed reference number articles (PMID) were identified. The search string was developed in consultation with an independent information expert (Dr Paul Braun (P.B.)) from the Central Medical Library (CMB) of the University Medical Center Groningen (UMCG). Once the algorithm for PubMed was finalised, the string was translated to PsychINFO and Excerpta Medica (EMBASE) in collaboration with P.B. All three databases were searched on 8 November 2021. Final search strings are given in Supplementary file 1.

### Screening

Results from the three databases were imported into EndNote 20.1 (Clarivate, Philadelphia, PA, USA; see https://support.clarivate.com/Endnote/s/article/Download-EndNote?language=en_US). Duplicate results were first detected with the ‘find duplicates’ feature in EndNote, and thereafter manually removed after review. The remaining results were imported into Rayyan (Rayyan, Cambridge, MA, USA; see www.rayyan.ai), and the duplicate removal was repeated. Next, an order of exclusion criteria was tested blindly on ten randomly selected papers to ensure synchronisation of the eligibility determination process between the two reviewers (see Supplementary file 7). After pilot testing the order, it did not require adjustments. The order was used for the double-blinded title/abstract and full-text screening. The outcomes of the double-blinded screening were discussed during weekly meetings. *A priori*, a third reviewer was identified (H.R.) in case any discrepancies could not be solved during those meetings. However, consultation of the third reviewer was not needed. Some disagreements between D.G.B. and S.H.B. arose from the different understanding of a clinical sample, resulting in a further specification of the definition of a clinical sample in the PROSPERO protocol (307987), as follows: ‘Studies with a small part of the sample being recruited from the general population (with diagnoses being established via interviews) and without information about mental healthcare, is accepted when at least a majority of the total sample is reported to receive mental health care’.

### Data collection

All data were extracted by means of an in-house developed data extraction form following the critical appraisal and data extraction for systematic reviews of prediction modelling studies (CHARMS) checklist.^18^ The data extraction form was piloted by taking a sample of five included studies. On 13 January 2022, data extraction was started. The first reviewer (D.G.B.) extracted all relevant data, and the second reviewer (S.H.B.) independently assessed data extraction validity using a random subsample of full texts (*n* = 6 out of 21), and S.H.B. reviewed the correctness of all reported outcomes of main interest, as shown in Table 3.

### Risk of bias and applicability

For each study, the risk of bias was assessed double-blinded by D.G.B. and S.H.B. using PROBAST. With PROBAST, the risk of bias was assessed in four domains: participants, predictors, outcomes and analyses.^10^ Each domain contained questions that should be rated with low, high or unclear concern. If one domain was judged to have a high risk of bias, the overall risk of bias was considered high. Equivalently, if one domain was assessed as unclear, the overall risk of bias was unclear. The evaluation of model applicability was judged using the same method of assessment. Potential publication bias of studies included in the meta-analysis was explored with a funnel plot using the R package metafor.^19^

### Data synthesis

Discrimination and calibration measures are of main interest, including the lowest and highest reported range if multiple models are externally validated on the same data-set and the type of measure reported. Other performance measures and descriptive data were also extracted and summarised from the papers: author(s), year of publication, source validation data-set (i.e. cohort, clinical trial), participant characteristics (i.e. type of disorder, age range), treatment characteristics (type of treatment, in- or out-patient setting, primary or secondary facility), outcome measures (definition, timing, method of assessment), predictors (psychosocial/clinical/biological nature), modelling methods (type of statistical analysis, method selection predictors), power (sample size, number of events per variable) and the handling of missing data.

### Predictor type categorisation

Predictor type categorisation is not straightforward, and transparent protocols for this are lacking in previous reviews in the field of precision psychiatry.^8,11^ As such, the qualitative method of grounded coding was applied. This qualitative method is directed towards finding common categories among codes and, if possible, higher-order categorisation may yield an overarching theme.^20^ To ensure transparency in reporting and possible reproduction, the resulting codebook generated by grounded coding is given in Supplementary file 6.

### Meta-analysis, sensitivity analyses and meta-regression

Formal meta-analysis to estimate an average discriminative ability is only useful if the criteria of the analysis are met.^13,21^ The data included in the meta-analysis are homogeneous on characteristics such as patients’ disorders, outcomes, predictors and study settings. However, the heterogeneity itself and its sources are informative for investigating how clinical and methodological aspects of the studies relate to their results.^14^

As such, a meta-analysis of omnibus discrimination performance measures was executed, as a prerequisite for the meta-regression. Any study that presented an omnibus measure of discrimination, such as the concordance statistic (c-statistic,^9,18^), or if not available, reported accuracy at single cut-off (ASC), was used in the meta-analysis. Given that ASC is less informative than the area under the curve (AUC),^22,23^ a sensitivity analysis was performed, excluding ASCs.

Currently, Cochrane^14^ does not provide recommendations for selecting models for meta-analysis in the case of multiple models reported within the same study. Therefore, we decided to follow the strategy of Lee et al,^24^ about using machine learning-based prediction models in psychiatry, to increase comparability. The best performing model per outcome was selected based on the highest reported discrimination. Multiple discrimination estimates from the same study were included under a few conditions as specified in the *a priori* established decision document (see Supplementary file 6).

When measures of uncertainty were missing for the c-statistic but the total sample size and the number of events were reported, these were estimated according to a formula described by Debray et al.^9^ Before the meta-analysis, discrimination measures were logit transformed to meet the normality assumptions. These transformations were done with the R package metamisc.^25^

For the assessment of the amount of heterogeneity, common parameters, *I*^2d^, tau^2e^ and the prediction interval between studies were used in the meta-analysis, which was implemented with the R package metafor.^19^ As heterogeneity between studies was anticipated, a random-effects model was used with restricted maximum likelihood. To ease interpretation, estimates were back transformed before being presented in tables and plots with the inverse logit transformation function (itrans.logit) of the metafor package, representing c-statistics (or ASCs).

To investigate which clinical and methodological aspects of the studies relate to the discriminative ability, a random-effects meta-regression was conducted. The number of investigated potential effect modifiers on model performance should be limited, as the likelihood of a false-positive result among subgroup analysis and meta-regression increases with the number of characteristics investigated.^18^ Following the Cochrane Handbook, potential effect modifiers may include study-level characteristics, such as population type, type of intervention or treatment, length of follow-up or methodology (i.e. design and quality). For testing interactions with categorical variables, the handbook advises that the number of studies should be minimally five per category.^14^ For testing interactions with continuous variables, the total number of studies should be minimally 10.^14^ The following study characteristics were eligible for meta-regression: type and number of predictors and type of disorder. The other above-mentioned characteristics could not be tested because of insufficient sample sizes. Regression coefficients, the associated *z*-scores, s.e. values, significance values and prediction intervals were reported as primary outcomes per moderator. The R script for these analyses is listed in Supplementary file 7.

## Results

### Study selection

**Fig. 1:**
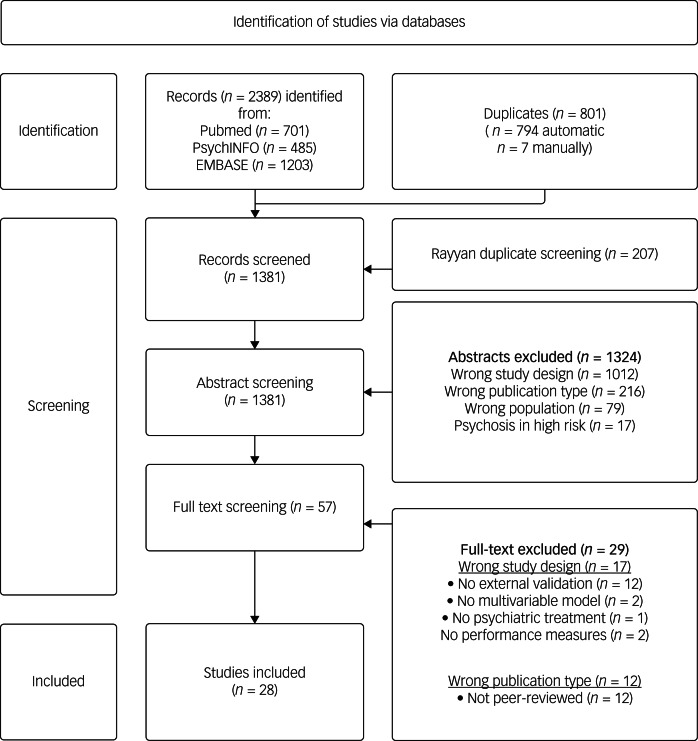
Preferred reporting items for systematic reviews and meta-analysis-conforming flowchart of the screening process. EMBASE, Excerpta Medica.

The systematic search in PubMed, EMBASE and PsycINFO yielded a total of 2389 hits. Fifty-seven studies were screened full text (see Fig. 1 for a flowchart of the screening process). In total, 28 studies^26–53^ were part of the systematic review (see Supplementary file 3 for the list of included papers).

The meta-analysis was conducted on the 21 studies^27,29,31–37,39,40,43–45^ that either included the needed parameters or those in which these parameters could be calculated to conform with the description in the analysis section. Notably, Ashar et al^26^ used a continuous metric for predictive accuracy and could not be included in the meta-analysis. Multiple models from the same study were included following the previously described decision document. Therefore, from the 21 studies, 28 models were included in the meta-analysis.

### Study characteristics

#### Source validation data-set

Five studies made use of data derived from registries, 14 studies included data from clinical trials (either open-label or randomised; nine and five, respectively), eight studies used observational cohort data and one study used survey data.

#### Participants and setting

Most studies focused on patients diagnosed with a mood disorder (unipolar *n* = 14, bipolar *n* = 2), followed by a mixed sample with severe mental disorders (SMIs) (*n* = 6) and psychotic disorders (*n* = 5), while there was one study that validated prediction models for anxiety and mood disorder separately. Most studies included adult patients only (*n* = 14), some included adolescents as well (*n* = 9) and in some papers, inclusion of adolescents remained unclear (*n* = 5). All study characteristics, including sample characteristics and care setting details, are given in Table 1. More details regarding the in- and exclusion criteria per validation data-set are listed in Supplementary file 7.

**Table 1:** Study characteristics

Reference	Disorder	Adolescents	Mean age	Treatment	Status	Setting	Outcome	Assessment	End-point
Ashar et al^26^	Anxiety	No	34.50	CBT	OUT	MHC (unspecified)	Symptom change	Self-report	16 weeks
Athreya et al^27^	Mood (DEP)	UNK	UNK	Antidepressant	OUT & in-patient status	All	Remission & response	INT	4 & 8 weeks
Athreya et al^28^	Mood (DEP)	UNK	ISPC: 41.80, Eli Lily: 46.50 and 42.00, MARS: 46.50	Antidepressant	OUT & in-patient status	UNK	Remission	INT	8 weeks
Bone et al^29^	SMI	Yes	41.19 (Sample1), 40.68 (Sample2)	Self-help therapy or psychotherapy	OUT	General MHC	Symptom change	Self-report	EOT
Cattaneo et al^30^	Mood (DEP)	No	39.00	Antidepressant	UNK	UNK	Response	INT	12 weeks
Chekroud et al^31^	Mood (DEP)	No	UNK	Antidepressant	OUT	GP & general MHC	Remission	Self-report	12 & 14 weeks
Fabbri et al^32^	Mood (DEP)	UNK	42.79 (STAR*D), 42.18 (GENDEP)	Antidepressant	OUT & in-patient status	GP & general MHC	TRS	INT	12 weeks
Fazel et al^33^	SMI	Yes	Der: 44.00	TAU	OUT	General MHC	Crime committance	OBS	1 year
Fazel et al^34^	SMI	Yes	UNK	TAU	OUT & in-patient status	All	Suicide	OBS	1 year
Fiedorowicz et al^35^	Mood (bipolar disorder)	No	35.50	TAU	OUT & in-patient status	MHC (unspecified)	Relapse	INT	1, 2, 3 & 5 years
Furukawa et al^36^	Mood (DEP)	No	UNK	Antidepressant	OUT	MHC (unspecified)	Remission	Self-report	1 & 3 weeks
Hayes et al^37^	Mood (bipolar disorder)	Yes	High risk: 41.07, low risk: 46.75	TAU (lithium)	OUT	GP	Side effects	OBS	5 years
Jha et al^38^	Mood (DEP)	No	42.60	Antidepressant	OUT	General MHC & specialised MHC	Remission & NMB	INT	8 weeks
Jha et al^39^	Mood (DEP)	No	UNK	Antidepressant	OUT	GP & general MHC	Remission	Self-report	12 weeks
Kambeitz-Ilankovic et al^40^	Psychosis	No	38.00 (11.84)–41.00 (11.84)^a^	ACT	OUT	MHC (unspecified)	Global functioning	INT	10 weeks
Kautzky et al^41^	Mood (DEP)	No	52.60	Antidepressant + TAU	OUT & in-patient status	Specialised MHC	TRS & response	INT	EOT
Klein et al^42^	Mood (DEP)	No	48.30	Antidepressant	OUT	All	Relapse	INT	3, 9, 15 & 24 months
Leighton et al^43^	Psychosis	UNK	24.64	TAU	OUT & in-patient status	General MHC	Remission & EET	INT	1 year
Leighton et al^44^	SMI	Yes	UNK	TAU	OUT & in-patient status	Specialised MHC	Remission	INT	6 months, 1 year
Nie et al^45^	Mood (DEP)	No	UNK	Antidepressant	OUT	UNK	TRS	INT	6 weeks
Nunez et al^46^	Mood (DEP)	No	35.40	Antidepressant + antipsychotic	OUT	Specialised MHC	Remission	Self-report	8 weeks
Ortiz et al^47^	Psychosis	Yes	30.10	Antipsychotic	OUT & in-patient status	Specialised MHC	TRS	INT	OUT: 4 weeks, in-patient status: discharge
Perlis et al^48^	Mood (bipolar disorder)	Yes	UNK	TAU	OUT	MHC (unspecified)	Adherence	Self-report	EOT
Perry et al^49^	Psychosis	Yes	24.45	TAU	OUT	General MHC	Side effects	OBS	6 years
Puntis et al^50^	SMI	Yes	26.70	TAU	OUT	General MHC	In-patient admission	OBS	1 year
Soldatos et al^51^	Psychosis	No	23.80	Antipsychotic	OUT & in-patient status	Specialised MHC	Remission	INT	6 weeks
Taliaz et al^52^	Mood (DEP)	UNK	37.60	Antidepressant	OUT	MHC (unspecified)	Response	INT	EOT
Wang et al^53^	Mood (DEP)	No	45.37	TAU	OUT	UNK	Relapse	INT	3 years

ACT, auditory-based computed tomography intervention; CBT, cognitive behavioural therapy; DEP, depressive disorders; Der, derivation sample; EET, employment, education or training status; EOT, end of treatment; GENDEP, genome-based therapeutic drugs for depression project; GP, general practitioner; INT, interview; ISPC, International SSRI Pharmacogenomics Consortium; MARS, Munich Antidepressant Response Signature project; MHC, medical hospital centre; NMB, no meaningful benefit; OBS, observational status; OUT, out-patient status; SMI, severe mental illness; STAR*D, Sequenced Treatment Alternatives to Relieve Depression study; TAU, treatment as usual; TRS, treatment resistance; UNK, unknown.

a. Calculated from data on training set and training and validation set.

#### Treatment outcome

Most studies reported clinical outcomes (*n* = 25), such as symptom change, remission status, adverse events and care consumption. One study^43^ reported a clinical (remission) and psychosocial outcome (employment, education and training status). One study described the psychosocial outcome of likelihood of crime committance.^34^ To assess the outcome, most studies used a clinician-rated instrument (*n* = 16) followed by self-report instruments (*n* = 7) and looking at event occurrence in registry data (*n* = 5). The timing of the end-points varied greatly. The earliest outcome assessment was at 1 week follow-up,^36^ while the latest was at 6 years follow-up.^49^ In some studies (*n* = 4) the outcome assessment depended on the end of treatment.^29,41,48,52^ A detailed description of the end-point assessment per study is given in Table 1.

#### Predictors

With the exception of three studies,^30,32,40^ all studies included clinical predictors in their model, such as symptom severity, past psychiatric history (PHX) or general medical history (GHX). Notably, studies that included psychosocial variables included biological variables as well (*n* = 16). Only a few studies (*n* = 7) included clinical predictors in their model(s). A detailed overview of the predictors used per study is given in Table 2. The codebook for coding which variable is considered which type is given in Supplementary file 7.

**Table 2:** (Type of) variables used per study

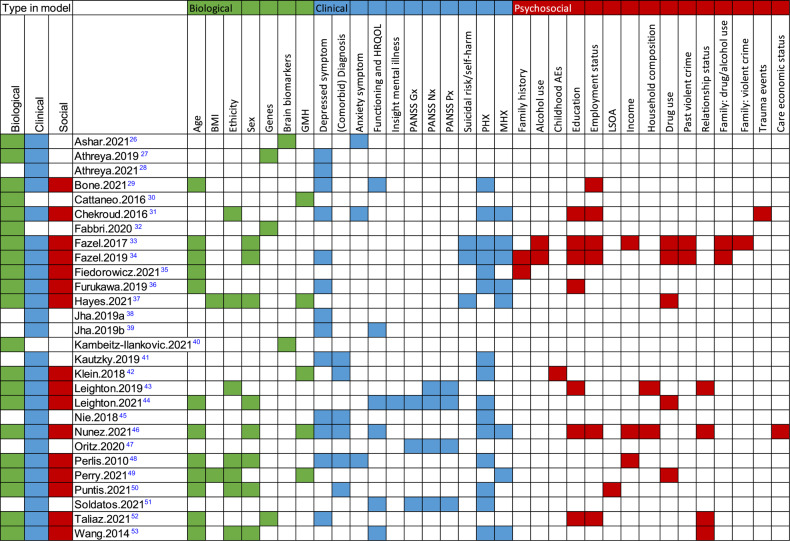

ASC, accuracy at single cut-off; AUC, area under the curve; BMI, body mass index; Childhood AEs, Childhood adverse events; GMH, general medical history; HRQOL, health-related quality of life; LSOA, lower super output area (measurement of neighbourhood deprivation); MHX, psychiatric medication history; NHS, National Health Service; PANSS Gx, positive and negative symptoms scale subscale general psychopathology; PANSS Nx, positive and negative symptoms scale subscale negative symptoms; PANSS Px, positive and negative symptoms scale subscale positive symptoms; PHX, psychiatric history and past psychiatric care use.

#### Performance measures

Out of the 28 included studies, 11 reported on both calibration and discrimination measures (see Table 3). The majority of studies only reported discrimination measures (*n* = 16). One study^30^ did not report omnibus calibration and discrimination measures, despite constructing and validating a clinical prediction model. The study reported accuracy measures instead (sensitivity, specificity). No studies included calibration measures only.

**Table 3:** Modelling methods and reported outcomes per study

Reference	*N*	Learning								Performance		Other^b^
		Stat	Machine learning	Model method	Missing data	EPV	Calibration measure	Performance	Discrimination measure	Lowest	Highest^a^	
Ashar et al^26^	42	X		Linear regression	LOCV	Not applicable	UNK	−	Predictive accuracy (ModR)	0.22 (NR)	−	
Athreya et al^27^	165–467		X	Random forest	CCA	UNK	UNK	−	ASC	0.66 (NR)	0.77 (NR)	Sens:0.80, Spec:0.71, NPV:0.62, PPV:0.85, NRI:0.69
Athreya et al^28^	33–237		X	Hidden Markov model	UNK	0.93–8.51	UNK	−	ASC	0.57 (NR)	0.86 (NR)	NRI:0.53
Bone et al^29^ Anx	923–18 580	X	X	Log, Bay, elastic net, XGBoost, SVM	UNK	UNK	UNK		AUC	0.527 (NR)	0.666 (NR)	
Bone et al^29^ Dep	923–18 580	X	X	Log, Bay, elastic net, XGBoost, SVM	UNK	UNK	UNK		AUC	0.60 (0.59–0.60)	0.81 (0.77–0.86)	PPV:0.79, NPV:0.69
Cattaneo et al^30^	68	X		LDF	UNK	11.50	UNK	−	UNK	−	−	Sens:1.00, Spec:1.00, PPV: 1.00, NPV:0.82
Chekroud et al^31^	134–51		X	GBM	CCA	2.72–2.88	UNK	−	ASC	0.51 (0.43–0.60)	0.60 (0.51–0.68)	Sens:0.56, Spec:0.63, NPV:0.60, PPV:0.60
Fabbri et al^32^	1343–965		X	GBM	Rem, Imp	UNK (218–571)	UNK	−	AUC	0.55 (0.51–0.59)	0.72 (0.58–0.86)	−
Fazel et al^33^	16 387	X		Log	Rem, Imp	13.75	UNK	−	C-stat	0.89 (0.85–0.93)	−	NRI:1.28, Sens:0.62, Spec:0.94, PPV:0.11, NPV:0.99
Fazel et al^34^	16 387	X		Log	Rem, Imp	8.18–10.55	Plot		C-stat	0.71 (0.66–0.75)		Sens:0.55, Spec:0.75, NPV:0.99, PPV:0.02, Brier:0.01, NRI:0.51
Fiedorowicz et al^35^ anypol	258		X	BCT	UNK	10.55	Plot	−	AUC	0.77 (0.77–0.79)	0.78 (0.77–0.79)^c^	−
Fiedorowicz et al^35^ Mania	258		X	BCT	UNK	10.55	UNK	−	AUC	0.72 (0.71–0.73)	0.72 (0.71–0.73)	−
Fiedorowicz et al^35^ MDB	258		X	BCT	UNK	10.55	UNK	−	AUC	0.81 (0.81–0.82)	0.82 (0.81–0.82)	−
Furukawa et al^36^	1002	X		Log	Imp	UNK	HLT	*P* = 0.29–0.41	AUC	0.73 (0.70–0.77)	0.82 (0.79–0.85)	−
Hayes et al^37^	137–934	X		Log	UNK	1.00–45.67	Plot	1.29	C-stat	0.85 (0.79–0.91)	0.89 (0.86–0.91)	ASC:0.81, Sens:0.86, Spec:0.80
Jha et al^38^	163	X		Log	CCA	8.25–20.25	Slope	<0.60	AUC	0.80 (NR)	0.84 (NR)	−
Jha et al^39^	399	X		Log	CCA	17.26–48.48	Plot	−	AUC	0.80 (0.75–0.84)	0.82 (0.77–0.87)	
Kambeitz-Ilankovic et al^40^	20		X	SVM	CCA	0.13	UNK	−	BAC	0.62.5	−	Sens:0.90, Spec:0.33, NPV:0.75, NND:4.10
Kautzky et al^41^	702	X		Log	CCA	UNK	UNK	−	ASC	0.87	−	Sens:0.86, Spec:0.88, NPV:0.92, PPV:0.79, FNR:0.12
Klein et al^42^	209	X		Log	Imp	16.09	Slope	0.56	AUC	0.59	−	−
Leighton et al^43^	64–7		X	Elastic net	Imp	2.38–4.20	UNK	−	AUC	0.63 (0.61–0.65)	0.88 (0.86–0.89)	Sens:0.82, Spec:0.88, NPV:0.88, PPV:0.82
Leighton et al^44^	399	X		Log	Imp	7.36	Slope	0.98 (0.85–1.11)	C-stat	0.73 (0.71–0.75)^c^	−	NRI:0.15
Nie et al^45^	225		X	Random forest, GBM, XGBoost, PLR, elastic net	UNK	UNK	UNK		AUC	0.6 (NR)	0.78 (0.70–0.86)^c^	ASC:0.84, Sens:1.00, Spec:0
Nunez et al^46^	180		X	Random forest, GBDT, XGBoost, PLR, elastic net	Imp	0.43–0.63	UNK	−	C-stat	0.65 (NR)	0.83 (0.76–0.90)^c^	Sens:0.81, Spec:0.67, NPV:0.92, PPV:0.44
Ortiz et al^47^	207	X		Log	UNK	27	UNK	−	AUC	0.76 (NR)	−	Sens:0.72, Spec:0.74
Perlis et al^48^	1869	X		Log	CCA	UNK	HLT	*P* < 0.01	ASC	0.81	−	Sens:0.19, Spec:0.90, NPV:0.89, PPV:0.21
Perry et al^49^	510	X		Log	Imp	10.75–14.33	Intercept	−0.02–−0.11	C-stat	0.74 (0.67–0.79)	0.75 (0.69−0.8)	*R*^2^:0.21 (0.18–0.25), Brier:0.07 (0.04–0.10)
Puntis et al^50^	1393	X		Log	Imp	9.88	Slope	0.86 (0.68–1.05)	AUC	0.70 (0.66–0.75)	−	NRI:0.20–0.40, Brier:0.09
Soldatos et al^51^	101		X	Elastic net	Imp	2.82	UNK	−	AUC	0.68 (0.56–0.79)	−	BAC:0.64, BNPV:0.61, BPPV:0.68
Taliaz et al^52^	132–251		X	SVM, XGBoost, random forest, AdaBoost	Rem, imp	UNK–97.94	UNK	−	BAC	0.61 (NR)	−	Sens:0.76, Spec:0.47, PPV:0.59, NPV:0.66
Wang et al^53^	1195	X		Log	Imp	13.35	HLT	3.51, *P* = 0.90	C-stat	0.72 (0.69–0.75)^c^	−	

AdaBoost, adaptive boosting; Anx, anxiety symptoms; Anypol, any polarity in bipolar; ASC, accuracy at single cutoff; AUC, area under the curve; BAC, balanced accuracy curve; Bay, Bayesian updating algorithm; BCT, boosted classification trees; Brier, Brier score; BNPV, Bayes negative predictive value; BPPV, Bayes positive predictive value; CCA, complete case analysis, C-stat, c-statistic; Dep, depressive symptoms; EPV, event per variable (lowest incidence category/number of predictors); FNR, false negative rate; GBDT, gradient boosting decision trees; GBM, gradient boosting; HLT, Hosmer–Lemeshow test; Imp, imputation; LDF, linear discriminant function; Learning, model learning type; LOCV, last observation carried forward; Log, Logistic regression; Mania, manic episode in bipolar; MDB, major depressive episode in bipolar; ModR, model-based *R* square; NND, number needed to diagnose; NR, not recorded; NRI, net reclassification index; NPV, net positive value; PLR, penalised linear regression; PPV, positive predictive value; Rem, removal; Sens, sensitivity; Spec, specificity; SVM, support vector machine; Stat, statistical learning; UNK, unknown, XGBoost, eXtreme gradient boosting.

All reported measures are rounded on two decimals.

a. Highest performance was only filled in if the study reported multiple models.

b. In case of multiple models, ‘other measures’ are given for the highest performing model.

c. Calculated confidence interval.

Out of 11 studies reporting calibration studies, most reported calibration slopes (*n* = 4), while some reported calibration plots (*n* = 3) or reported results from the Hosmer–Lemeshow test (*n* = 3). One study reported the calibration intercept. Studies that included discrimination measures (*n* = 27) reported AUC statistics (*n* = 14), c-statistics (*n* = 7), ASCs (*n* = 5) and a continuous outcome with an *R*^2^ measure to represent predictive accuracy (*n* = 1).

#### Modelling methods

Almost all studies constructed classification models (*n* = 27). Many studies (see Table 3) constructed statistical learning models (*n* = 16), with logistic models being the most prevalent (*n* = 14). A total of 11 models were constructed using machine learning only, with some form of boosting (either gradient, extreme or adaptive) (*n* = 9) and elastic net (*n* = 4) the most utilised techniques. One study^29^ used both statistical and machine learning methods. For further detail, see Supplementary file 7, in which a detailed description of the utilised technique per study is listed.

#### Handling of missing data

Several studies (*n* = 9) accounted for missing data by imputation only. A few studies (*n* = 4) excluded variables from analysis if a given share of a variable was missing and, subsequently, imputed variables. Notably, some studies (*n* = 7) relied on complete case analysis. One study^26^ applied last observation carried forward for handling missing data. In various studies (*n* = 7), handling of missing data remained unclear from the text and supplement.

#### Risk of bias

Following PROBAST, the overall quality of reporting in the studies was low (see Supplementary file 6 for study-specific (sub)domain evaluations). Main areas of high concern with respect to bias introduction were the poor reporting on the handling of missing data, analyses and outcomes.

#### Applicability

Applicability was also assessed with PROBAST. Main areas of concern were participant in- and exclusion criteria, included predictors and outcome assessment. Many studies were rated as high concern on the participant domain, mostly because of stringent in- and exclusion criteria, which harmed the generalisability of the model (*n* = 12). Seven studies were rated as of high concern on predictor feasibility as operation of the chosen predictors in clinical practice was challenging. For example, one study^26^ included the predictor amygdala connectivity, which was calculated from a functional magnetic resonance imaging (fMRI) scan. Following the PROBAST guidelines, this study is rated of high concern with regards to applicability. Not only are neuroimaging data-sets prone to variability in analysis,^54^ but the limited sample size of these studies also limits the applicability of the results.^55,56^ The specialised techniques required for imaging techniques and their associated complexity and cost may make the applicability lower for widespread clinical deployment.

A few studies (*n* = 2) were rated as of high concern on the outcome feasibility for various reasons. Notably, only two studies^36,50^ were of low concern in both risk of bias and applicability.

### Meta-analysis

In total, 28 models (derived from 21 studies) were eligible for meta-analysis according to the pre-specified criteria. The funnel in Supplementary file 5 indicates heterogeneity in the discrimination performance, even between studies with a small standard error.

**Fig. 2:**
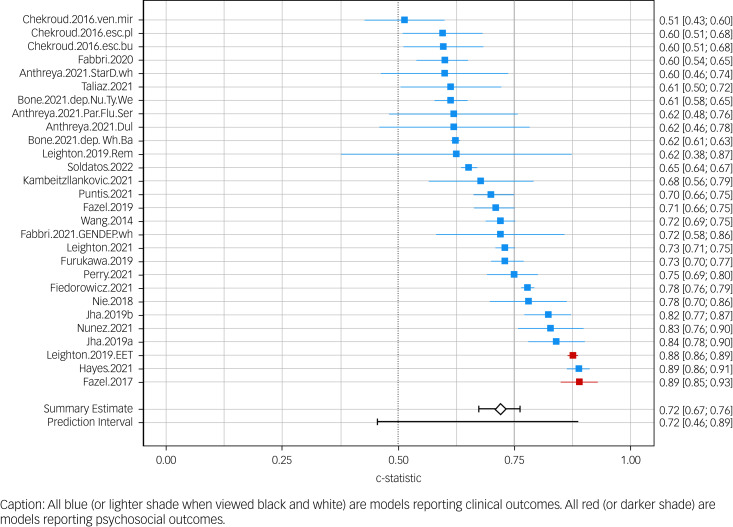
Forest plot of all eligible models (*n* = 28). Includes reported ASC and AUC. For a sensitivity analysis with only AUC, please refer to Supplementary file 4. ASC, accuracy at single cut-off; AUC, area under the curve; Bu, buproprion; dul, duloxetine; EET, Education Employment Training status; esc, escitalopram; flu, fluoxetine; mir, mirtazapine; NHS, National Health Service; Nu.Ty.We, Cumbria Northumberland Tyne and Wear NHS Foundation Trust; par, paroxetine; pl, placebo; Rem, remission; ser, sertraline; ven, venlafaxine; wh, whole; Wh.Ba, Whittington Barnet Enfield Haringey Pennine and Humber NHS Foundation Trust.

As expected, the heterogeneity among studies was substantial (*I*^2^ = 97.83%, tau^2^ = 0.28). Overall, evidence for the performance accuracy was mixed. The overall summary discrimination performance was fair (>0.70; point estimate: 0.72; 95% CI [0.46; 0.89]). However, the wide prediction interval (see Fig. 2) indicates that some models performed no better than chance (confidence interval surpasses the value 0.50). Excluding the ASC-reporting studies resulted in a slightly higher summary estimate and narrower confidence interval: 0.76 [0.71–0.80]. The prediction interval surpassed the threshold of 0.50, meaning that all included models performed better than chance [0.51; 0.90]. The forest plot of this sensitivity analysis is presented in Supplementary file 4.

As can be viewed in Fig. 2, the two studies that reported a psychosocial outcome performed among the best. However, because of the low sample size in this category, no meta-regression on the type of outcome could be conducted.

#### Potential sources of heterogeneity

When applying the criteria given in the Method section, the following sources of heterogeneity could be investigated: type of disorder and number and type of included predictors.

#### Type of disorder

Diagnosis of a depressive disorder was a significant moderator (*B* = −0.47, *z* = −2.31, s.e. = 0.20 *P* = 0.02, prediction interval = −0.86; −0.07) and it explained 3.41% of the residual heterogeneity. Models that predict outcomes for individuals diagnosed with depressive disorders (*n* = 18) showed lower discrimination than models that predict outcomes for other disorders (*n* = 10). The remaining models included SMIs, such as psychosis (*n* = 6) and bipolar disorder (*n* = 2), or mixed samples (*n* = 2).

#### Number of included predictors

The number of included predictors did not significantly moderate discrimination performance (*B* = 0.00, *z* = 0.25, s.e. = 0.01 *P* = 0.80, prediction interval = −0.01; 0.01).

#### Type of predictors

Out of 28 eligible models, 18 models used biological, psychosocial and clinical predictors. The usage of a combination of these three categorical variables did not significantly moderate the discrimination performance (*B* = 0.11, *z* = 0.46, s.e. = 0.24, *P* = 0.65, prediction interval = −0.35; 0.57). The use of clinical predictors (*n* = 7) only did not significantly moderate discrimination performance (*B* = 0.03, *z* = 0.13, s.e. = 0.26, *P* = 0.89, prediction interval = −0.48; 0.55). As there were few models using biological predictors exclusively (*n* = 2), a meta-regression for biological models was not performed. There were no models using psychosocial predictors only.

## Discussion

This is the first systematic review specifically examining the performance and applicability of externally validated prediction models that estimate treatment outcomes for individuals diagnosed with a mood, anxiety or psychotic disorder. Twenty-eight studies were identified. Studies included in this review differed by source of validation data-set, participant in- and exclusion criteria, in definition and in timing of the assessment method, treatment outcome, type and number of included predictors, reporting of discrimination and calibration measures, handling of missing data and the modelling methods. The majority of the studies were labelled as high risk of bias by the PROBAST, because of methodological concerns, (poor reporting of) analyses or inappropriate handling of missing data. The applicability of the included studies was overall poor because of strict in- and exclusion criteria, included predictors (feasibility to obtain predictors within a clinical context) and outcome assessment. The two studies scoring of low concern with regards to risk of bias and applicability^36,50^ shared the following characteristics: they were both pragmatic in nature (either trial close to clinical practice or routinely collected data), predicted a clinical outcome, utilised accessible clinical, biological and psychosocial variables, employed logistic regression and imputed missing data using chained equations (see Supplementary Table 7.6).

In the meta-analysis, the overall summary discrimination measure was fair. Nevertheless, some models had such wide prediction intervals that they should be interpreted as performing on chance level, while other models were adequate. The two models reporting psychosocial outcomes performed among the best. Out of the meta-regressions performed, only the type of disorder (depression versus other disorders) explained a limited share of the heterogeneity; models for participants with depression performed worse than others.

Despite the effort to conduct a comprehensive search and include all relevant literature, there may have been papers omitted that should have been included by the way we conducted our title and abstract screening. As an example, during the screening of titles and abstracts, we excluded a paper on lithium response by Nunes et al^57^ (2020) because the methods in the abstract mentioned cross-validation as the validation method, while the full text also included a form of external validation (leave-one-site-out). However, we had to keep a balance between rigorousness and effort.

Because of resource constraints, there was a partial duplication in data extraction. For full texts, only a subsample was taken, and while every effort was made to ensure accuracy, it is possible that minor discrepancies may have occurred. It is important to note that all outcomes of primary interest, particularly those outlined in Table 3, were meticulously double-checked. Despite our best efforts, we acknowledge the possibility of minor discrepancies in other areas.

### Literature comparison and interpretation

A recent meta-review^58^ identified four externally validated models, of which one was a predictor-finding study according to the review of Lee et al.^24^ A systematic review and meta-analysis for clinical prediction models above and beyond psychiatric treatment outcomes in psychiatry^11^ identified 25 externally validated predictive models. The number of studies identified in the current review illustrates a rapidly growing interest in the field of psychiatry with regards to clinical prediction model research. Still, the number of externally validated models is very low compared to the number of developed models. In the meta-review of Gillet et al,^58^ the lack of external validation in the literature was noted. In the review of Lee et al^24^ and Salazar de Pablo et al,^11^ only 20.0% and 29.9% of the included studies externally validated their models, respectively. Synthesising the finding of relatively few externally validated models compared to the number of developed models, literature comparison supports our notion that the field could greatly benefit from a shift in focus from developing new models to external validation of developed models.

The finding that there was large methodological heterogeneity among studies constructing and validating clinical prediction models is in line with the conclusions of the studies of Gillet et al^58^ and Salazar de Pablo et al.^11^ More specifically, Gillet et al^58^ concluded that within systematic reviews, definition of treatment response, predictor variables and assessment thereof varied greatly among studies, and Salazar de Pablo et al^11^ concluded that the main limitation of their study was the heterogeneity of characteristics of prediction models. Chekroud et al^59^ highlight the challenge of external validation across clinical trials, thereby advocating for the importance of identifying trial-level characteristics in relation to patient outcomes to improve model generalisability. As the growth of mental healthcare delivery systems enables extensive data collection, longitudinal validation methods are the most pragmatic form of examining a model's generalisability.

Similar to previous work by Salazar de Pablo et al,^11^ Gillet et al^58^ and Meehan et al,^8^ the majority of the externally validated prediction models were classification models. One could speculate why this is the case. Perhaps, for clinical decision-making, the classification of treatment outcomes into binary categories may be considered more clinically useful than a change in symptom score. In our review, several studies employed a change in or a threshold on symptom scores to determine treatment outcome status such as remission versus non-remission or response versus non-response. Another possible reason for only finding classification models could be inherent to the search string used. If calibration and discrimination measures are underreported in non-classification models, they are not included in the review.

We extend previous work by Salazar de Pablo et al,^11^ as the frequent use of clinical variables and the less frequent use of biomarkers (such as genetic information) is identified in this review as well. Salazar de Pablo et al^11^ found that most models were based on clinical predictors, and that there is no evidence that models that include biomarkers outperform other types of models. In existing biomarker-based models, the great variability in raw data processing and feature type selection potentially hampers successful external validation.^54,55^ Some even question if biomarkers are a necessity to include when estimating treatment outcomes. In Meehan et al,^8^ studies reporting models with a majority of biomarkers were excluded because of pragmatic concerns. In this review, no such restrictions were applied. Notably, still few externally validated models were identified that predominantly used biomarkers, and were of poor performance. This supports the notion that biomarkers are unable or not yet sufficiently able to reflect psychopathological mechanisms.

Notably, we could not identify externally validated clinical prediction models that contained tests of known psychological mechanisms. For example, the role of negative bias (i.e. the tendency to pay more attention to negative information) is a well-established characteristic in the psychopathology of patients suffering from depressive disorders, anxiety disorders, bipolar disorders and schizophrenia,^33–35^ of which severity can be assessed by the dot-probe computer task.^36^ In the review of Lee et al,^24^ a study constructed a prediction model utilising a cognitive-emotional biomarker, but unfortunately, this model was not externally validated. Based on this research, it is tempting to speculate that models using outcomes of cognitive tests fail to replicate in external validation, or that this observation identifies a promising gap in the literature that invites further exploration.

Comparing accuracy performance among reviews must be performed with great caution, as the reported accuracy interval depends on the type of validation the included models were tested with.^13,21^ In this review, only externally validated models were included, regardless of the model's performance in the development data-set (see Supplementary Table 7.7). Based on the lower point-estimates and wider prediction intervals of this study, one could conclude that the accuracy performance in the current study is lower compared to the accuracy of the reported prediction models of Salazar de Pablo et al.^11^ This is also observed in the review of Meehan et al.^8^ However, in both reviews, the sample in the meta-analyses consisted mostly of internally validated models, making the higher reported discrimination measures a more likely finding.^9,13^ Moreover, in both reviews, a protocol for the selection of models for meta-analysis was lacking.

Comparing the findings of systematic reviews and meta-analysis enhances the risk of ecological fallacy, as aggregated data can mask important differences between subgroups or individuals, which is contradictory to the mission of precision psychiatry. One may even argue that a narrative review may be more appropriate, as this publication type allows for a broader scope, broader audience and more flexibility.^60^ However, narrative reviews are, because of their flexibility, more prone to bias in the gathering and evaluation of evidence.^15^

This review illustrates the symbiotic relationship between introduced bias and applicability concern because of methodological design. In our review, only two out of 28 models were both of low concern when considering risk of bias and applicability. The reviews of Meehan et al^8^ and Salazar de Pablo et al^11^ identified key weaknesses in model construction and performance: underpowered studies, failure to report key aspects of model handling (missing data handling, predictive performance (mostly calibration) and bias-prone variable selection strategies. Not surprisingly, most models in both reviews were rated of high concern in both the risk of bias and applicability domains.

The observation, informed by the meta-regression, that models predicting depressive treatment outcomes performed worse compared to the other models in our sample is novel and intriguing. A possible explanation to consider is the particular case mix of the ‘rest’ category in the meta-regression, which included mainly SMI samples. Studies using samples with more severe disorders are more often conducted in patients treated in secondary care, and may have different distributions of outcomes and predictors, which may lead to different accuracy performance.^21^ Relatedly, the presentation of patients with moderate severity, which is often the case for depression, is typically more heterogeneous, thereby making it more difficult for a model to perform well as compared to in a sample with more homogeneous severely mentally ill patients.^13^ Notably, the models predicted psychosocial outcomes for a SMI population, raising the question whether the good discrimination of these models is due the sample, the type of outcome predicted or both.

The encountered diversity of studies and settings in this review reflects the challenge of clinical model implementation. The finding that many models were labelled of ‘high concern’ with regard to the risk of bias and/or applicability indicates that few models seem ready for further implementation in clinical practice to aid treatment allocation. For the implementation of clinical prediction models into practice, the need for close examination of the clinical setting before model implementation remains, regardless of whether they have been externally validated. The observation in the meta-regression that depression models reported lower accuracy, and visually apparent stronger accuracy for psychosocial outcomes in the meta-analysis, are new and highly remarkable. More research is needed to understand these differences and their implications.

In conclusion (large-scale) implementation of individualised externally validated prediction models in clinical practice does not seem to be feasible in the near future. Few models seem ready for further implementation in clinical practice to aid treatment allocation. Besides the need for more external validation studies, we recommend close examination of the clinical setting before model implementation.

## Supporting information

Burghoorn et al. supplementary material 1 Burghoorn et al. supplementary material

Burghoorn et al. supplementary material 2 Burghoorn et al. supplementary material

Burghoorn et al. supplementary material 3 Burghoorn et al. supplementary material

Burghoorn et al. supplementary material 4 Burghoorn et al. supplementary material

Burghoorn et al. supplementary material 5 Burghoorn et al. supplementary material

Burghoorn et al. supplementary material 6 Burghoorn et al. supplementary material

Burghoorn et al. supplementary material 7 Burghoorn et al. supplementary material

## Data Availability

Data availability is not applicable to this article as no new data were created or analysed in this study.

## Footnotes

a Definition of discrimination:^9^ ‘a model's ability to discriminate between patients who will benefit and who will not benefit from a given treatment’.

b Definition of calibration:^9^ ‘a model's ability to accurately predict estimated outcomes compared to observed outcomes’.

c Following the TRIPOD statement,^13^ reporting calibration and/or discrimination measures, or at least accuracy at a single cut-off.

d *I*^2^: estimate of the proportion of variability meta-analysis that is explained by methodological differences rather than sampling error.

e Tau^2^: estimate of the standard deviation of the true effect sizes, used to calculate the prediction interval.
