# Supplementary material for: Externally validated clinical prediction models for estimating treatment outcomes for patients with a mood, anxiety or psychotic disorder: systematic review and meta-analysis
Source: BJPsych Open. 2024 Dec 5;10(6):e221. doi: 10.1192/bjo.2024.789 (PMC11698186; doi:10.1192/bjo.2024.789)
Supplement: Burghoorn et al. supplementary material 1 — Burghoorn et al. supplementary material [file S2056472424007890sup001.docx]

| Supplement 2: PICOT table | |
| --- | --- |
| Component | Description |
| Population | Adult and elderly patients with mood (including anxiety), bipolar, and psychotic disorders in mental health care treatment. Studies with adolescents included are allowed if they are part of a larger sample including adults and/or elderly.  Exclusion:  Studies including children (under the age of 12)  Studies focused exclusively on adolescent populations (under the age of 18).  Studies focused exclusively on postnatal mental disorders (e.g. postnatal depression or anxiety)  Studies including individuals recruited from the general population (not from mental health care)  Studies including individuals without diagnosis (no interview or information from patient file). |
| Intervention(s) | Externally validated multivariable prediction (predictive) models for estimating any type of treatment outcome in mental health care.  Exclusion:  Studies that do not report discrimination/classification or calibration measures  Studies predicting the prevention or onset of new disorders |
| Comparator(s)/control | The comparator condition will be individuals who do not develop the specific outcome of interest. |
| Outcome | *Quantitative:* Performance of prediction models, as assessed with discrimination and calibration from the externally validated model. Externally validated models are included, regardless of whether the paper also includes the development of the model (if not included, the development paper needs to be findable).   - Discrimination: The c-index or area-under-the-curve (or, where both are unavailable, measures of sensitivity, specificity, or model accuracy at a single risk cut-off) - Calibration: Presented calibration plot and/or constituent statistics (intercept, slope), or the Hosmer-Lemeshow goodness-of-fit test. - Other types of performance measures , e.g. Positive Predictive Value and net reclassification improvement. - For the meta-regression, the outcome is a measure of accuracy. This can be the c-index, the area-under-the curve or some other variant.   *Qualitative:*   - CHARMS-compliant data extraction of relevant study characteristics - Overview of the type of predictors used in these externally validated models categorized by biological, psychological, clinical or social nature. - Risk of bias and applicability concerns as measured with the PROBAST tool |
| Type of study | Observational cohort studies, registry data and secondary analysis of randomized controlled trials with datasets predominantly containing a clinical population, both in the construction and validation dataset. |
