# Supplementary material for: Externally validated clinical prediction models for estimating treatment outcomes for patients with a mood, anxiety or psychotic disorder: systematic review and meta-analysis
Source: BJPsych Open. 2024 Dec 5;10(6):e221. doi: 10.1192/bjo.2024.789 (PMC11698186; doi:10.1192/bjo.2024.789)
Supplement: Burghoorn et al. supplementary material 2 — Burghoorn et al. supplementary material [file S2056472424007890sup002.pdf]

## Supplement 3 – List of all included papers in the review


Ashar YK, Clark J, Gunning FM, Goldin P, Gross JJ, Wager TD. Brain markers predicting response to cognitive-behavioral therapy for social anxiety disorder: an independent replication of Whitfield-Gabrieli et al. 2015. Transl Psychiatry. 2021;11(1):260.

Athreya AP, Brückl T, Binder EB, John Rush A, Biernacka J, Frye MA, et al. Prediction of short-term antidepressant response using probabilistic graphical models with replication across multiple drugs and treatment settings. Neuropsychopharmacology. 2021;46(7):1272-82.

Athreya AP, Neavin D, Carrillo-Roa T, Skime M, Biernacka J, Frye MA, et al. Pharmacogenomics-Driven Prediction of Antidepressant Treatment Outcomes: A Machine-Learning Approach With Multi-trial Replication. Clin Pharmacol Ther. 2019;106(4):855-65.

Bone C, Simmonds-Buckley M, Thwaites R, Sandford D, Merzhvynska M, Rubel J, et al. Dynamic prediction of psychological treatment outcomes: development and validation of a prediction model using routinely collected symptom data. Lancet Digit Health. 2021;3(4):e231-e40.

Cattaneo A, Ferrari C, Uher R, Bocchio-Chiavetto L, Riva MA, Pariante CM. Absolute Measurements of Macrophage Migration Inhibitory Factor and Interleukin-1-β mRNA Levels Accurately Predict Treatment Response in Depressed Patients. Int J Neuropsychopharmacol. 2016;19(10).

Chekroud AM, Zotti RJ, Shehzad Z, Gueorguieva R, Johnson MK, Trivedi MH, et al. Cross-trial prediction of treatment outcome in depression: a machine learning approach. Lancet Psychiatry. 2016;3(3):243-50.

Fabbri C, Kasper S, Kautzky A, Zohar J, Souery D, Montgomery S, et al. A polygenic predictor of treatment-resistant depression using whole exome sequencing and genome-wide genotyping. Transl Psychiatry. 2020;10(1):50.

Fazel S, Wolf A, Larsson H, Lichtenstein P, Mallett S, Fanshawe TR. Identification of low risk of violent crime in severe mental illness with a clinical prediction tool (Oxford Mental Illness and Violence tool [OxMIV]): a derivation and validation study. Lancet Psychiatry. 2017;4(6):461-8.

Fazel S, Wolf A, Larsson H, Lichtenstein P, Mallett S, Fanshawe TR. The prediction of suicide in severe mental illness: development and validation of a clinical prediction rule (OxMIS). Transl Psychiatry. 2019;9(1):98. https://doi.org/10.1038/s41398-019-0428-3

Fiedorowicz JG, Merranko JA, Iyengar S, Hower H, Gill MK, Yen S, et al. Validation of the youth mood recurrences risk calculator in an adult sample with bipolar disorder. J Affect Disord. 2021;295:1482-8.

Furukawa TA, Kato T, Shinagawa Y, Miki K, Fujita H, Tsujino N, et al. Prediction of remission in pharmacotherapy of untreated major depression: development and validation of multivariable prediction models. Psychol Med. 2019;49(14):2405-13.

Hayes JF, Osborn DPJ, Francis E, Ambler G, Tomlinson LA, Boman M, et al. Prediction of individuals at high risk of chronic kidney disease during treatment with lithium for bipolar disorder. BMC Med. 2021;19(1):99.

Jha MK, Minhajuddin A, South C, Rush AJ, Trivedi MH. Irritability and Its Clinical Utility in Major Depressive Disorder: Prediction of Individual-Level Acute-Phase Outcomes Using Early Changes in Irritability and Depression Severity. Am J Psychiatry. 2019;176(5):358-66.

Jha MK, South C, Trivedi J, Minhajuddin A, Rush AJ, Trivedi MH. Prediction of Acute-Phase Treatment Outcomes by Adding a Single-Item Measure of Activity Impairment to Symptom Measurement: Development and Validation of an Interactive Calculator from the STAR*D and CO-MED Trials. Int J Neuropsychopharmacol. 2019;22(5):339-48.

Kambeitz-Ilankovic L, Vinogradov S, Wenzel J, Fisher M, Haas SS, Betz L, et al. Multivariate pattern analysis of brain structure predicts functional outcome after auditory-based cognitive training interventions. NPJ Schizophr. 2021;7(1):40.

Kautzky A, Dold M, Bartova L, Spies M, Kranz GS, Souery D, et al. Clinical factors predicting treatment resistant depression: affirmative results from the European multicenter study. Acta Psychiatr Scand. 2019;139(1):78-88.

Klein NS, Holtman GA, Bockting CLH, Heymans MW, Burger H. Development and validation of a clinical prediction tool to estimate the individual risk of depressive relapse or recurrence in individuals with recurrent depression. J Psychiatr Res. 2018;104:1-7.

Leighton SP, Krishnadas R, Chung K, Blair A, Brown S, Clark S, et al. Predicting one-year outcome in first episode psychosis using machine learning. PLoS One. 2019;14(3):e0212846.

Leighton SP, Krishnadas R, Upthegrove R, Marwaha S, Steyerberg EW, Gkoutos GV, et al. Development and Validation of a Nonremission Risk Prediction Model in First-Episode Psychosis: An Analysis of 2 Longitudinal Studies. Schizophr Bull Open. 2021;2(1):sgab041.

Nie Z, Vairavan S, Narayan VA, Ye J, Li QS. Predictive modeling of treatment resistant depression using data from STAR*D and an independent clinical study. PLoS One. 2018;13(6):e0197268.

Nunez JJ, Nguyen TT, Zhou Y, Cao B, Ng RT, Chen J, et al. Replication of machine learning methods to predict treatment outcome with antidepressant medications in patients with major depressive disorder from STAR*D and CAN-BIND-1. PLoS One. 2021;16(6):e0253023.

Ortiz BB, Higuchi CH, Noto C, Joyce DW, Correll CU, Bressan RA, et al. A symptom combination predicting treatment-resistant schizophrenia - A strategy for real-world clinical practice. Schizophr Res. 2020;218:195-200.

Perlis RH, Ostacher MJ, Miklowitz DJ, Hay A, Nierenberg AA, Thase ME, et al. Clinical features associated with poor pharmacologic adherence in bipolar disorder: results from the STEP-BD study. J Clin Psychiatry. 2010;71(3):296-303.

Perry BI, Osimo EF, Upthegrove R, Mallikarjun PK, Yorke J, Stochl J, et al. Development and external validation of the Psychosis Metabolic Risk Calculator (PsyMetRiC): a cardiometabolic risk prediction algorithm for young people with psychosis. Lancet Psychiatry. 2021;8(7):589-98.

Puntis S, Whiting D, Pappa S, Lennox B. Development and external validation of an admission risk prediction model after treatment from early intervention in psychosis services. Transl Psychiatry. 2021;11(1):35.

Soldatos RF, Cearns M, Nielsen M, Kollias C, Xenaki LA, Stefanatou P, et al. Prediction of Early Symptom Remission in Two Independent Samples of First-Episode Psychosis Patients Using Machine Learning. Schizophr Bull. 2022;48(1):122-33.

Taliaz D, Spinrad A, Barzilay R, Barnett-Itzhaki Z, Averbuch D, Teltsh O, et al. Optimizing prediction of response to antidepressant medications using machine learning and integrated genetic, clinical, and demographic data. Transl Psychiatry. 2021;11(1):381.

Wang JL, Patten S, Sareen J, Bolton J, Schmitz N, MacQueen G. Development and validation of a prediction algorithm for use by health professionals in prediction of recurrence of major depression. Depress Anxiety. 2014;31(5):451-7.
