# Supplementary material for: Externally validated clinical prediction models for estimating treatment outcomes for patients with a mood, anxiety or psychotic disorder: systematic review and meta-analysis
Source: BJPsych Open. 2024 Dec 5;10(6):e221. doi: 10.1192/bjo.2024.789 (PMC11698186; doi:10.1192/bjo.2024.789)
Supplement: Burghoorn et al. supplementary material 3 — Burghoorn et al. supplementary material [file S2056472424007890sup003.pdf]

Supplement 4 – Additional sensitivity analysis

Title: Additional sensitivity meta-analysis, excluding models reporting discrimination by accuracy at single cut-off (ASC).

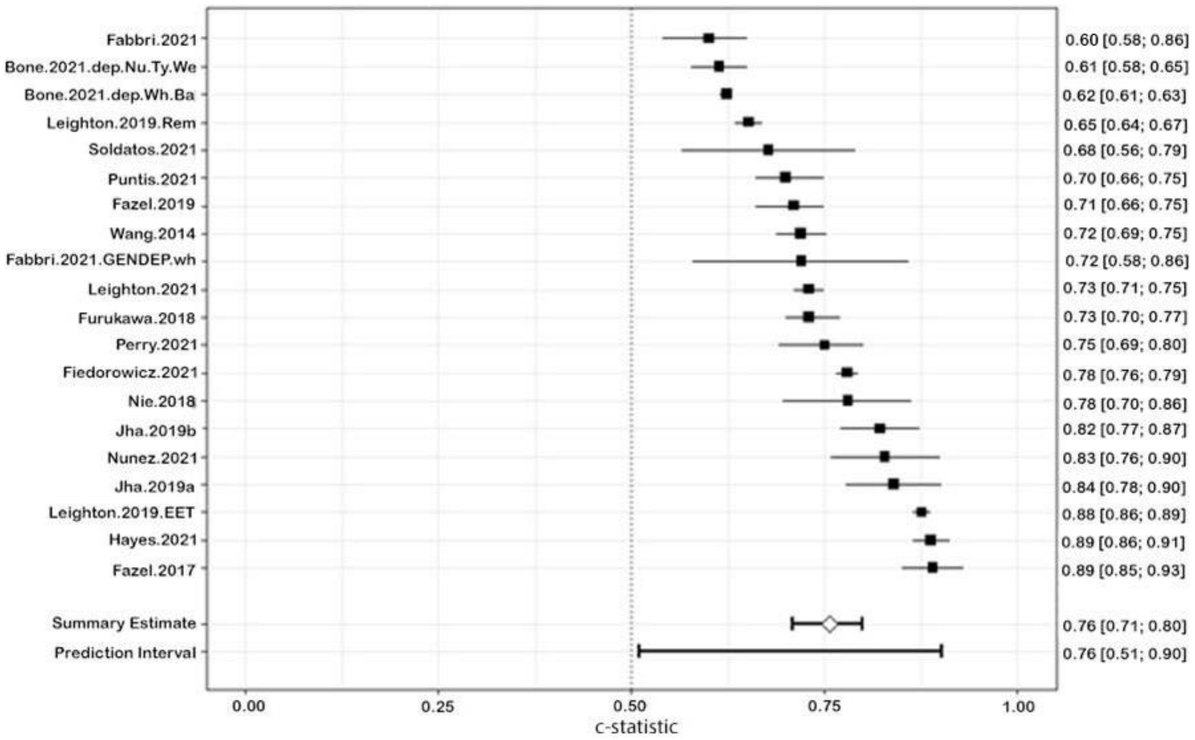

Caption: The overall prediction interval ranges from 0.51 to 0.90; thereby surpassing the c-statistic threshold of 0.50, meaning that all models that did not report ASC performed better than chance.
