# Supplementary material for: Externally validated clinical prediction models for estimating treatment outcomes for patients with a mood, anxiety or psychotic disorder: systematic review and meta-analysis
Source: BJPsych Open. 2024 Dec 5;10(6):e221. doi: 10.1192/bjo.2024.789 (PMC11698186; doi:10.1192/bjo.2024.789)
Supplement: Burghoorn et al. supplementary material 4 — Burghoorn et al. supplementary material [file S2056472424007890sup004.pdf]

Supplement 5 – Publication bias

**Title: Funnel plot of included studies in systematic review**

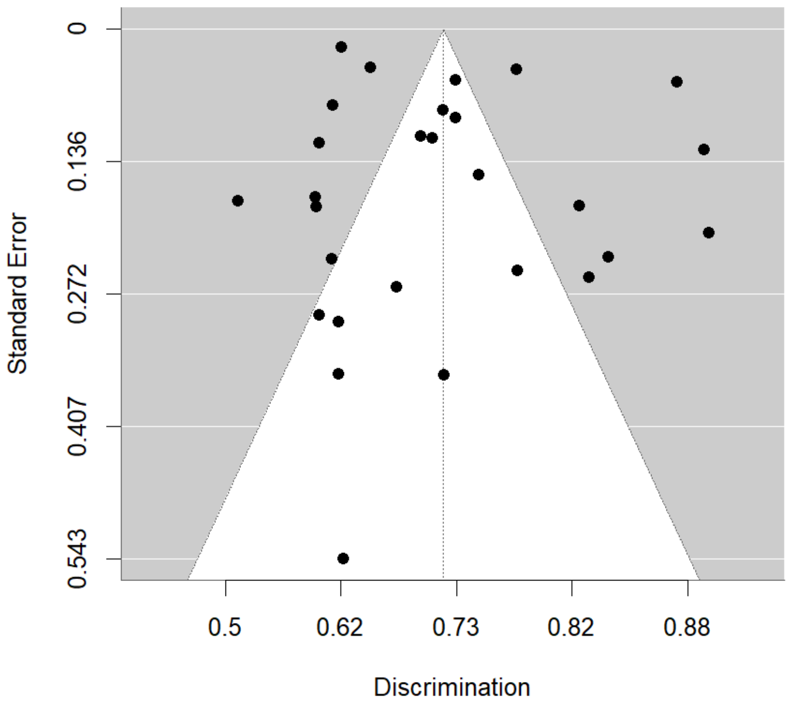

Caption: the dot close to the x-axis represents one study (42) with a large standard deviation suggesting that there is one lower-powered study included in the meta-analysis. Despite the symmetrical concentration of dots at the top, the funnel plot indicates substantial variance in discrimination performance among high-powered studies.
