# Supplementary material for: Externally validated clinical prediction models for estimating treatment outcomes for patients with a mood, anxiety or psychotic disorder: systematic review and meta-analysis
Source: BJPsych Open. 2024 Dec 5;10(6):e221. doi: 10.1192/bjo.2024.789 (PMC11698186; doi:10.1192/bjo.2024.789)
Supplement: Burghoorn et al. supplementary material 5 — Burghoorn et al. supplementary material [file S2056472424007890sup005.pdf]

Supplement 6 – Quality assessment

6.1 Reproducibility - abstract screening order

**Title: applied abstract and full-text screening order**	
1	Original data (exclusion = no original data)/ wrong publication type
2	Peer-reviewed (exclusion = not peer-reviewed)/wrong publication type
3	External validation of prediction model (exclusion = no external validation of prediction model)/wrong study design
4	Mood, anxiety or psychotic disorder (exclusion = wrong or no disorder)/wrong study population
5	Prediction of psychiatric treatment outcome (exclusion = no psychiatric treatment outcome predicted)/wrong study design
6	Including adults or elderly (exclusion = no adults or elderly included)/wrong study population
7	No postnatal condition exclusively (exclusion = postnatal psychiatric condition)/wrong study population
**Caption:** applied abstract and full-text screening order	

6.2 Reproducibility – Decision chart inclusion models meta-analysis

Title: decision chart inclusion models into the meta-analysis

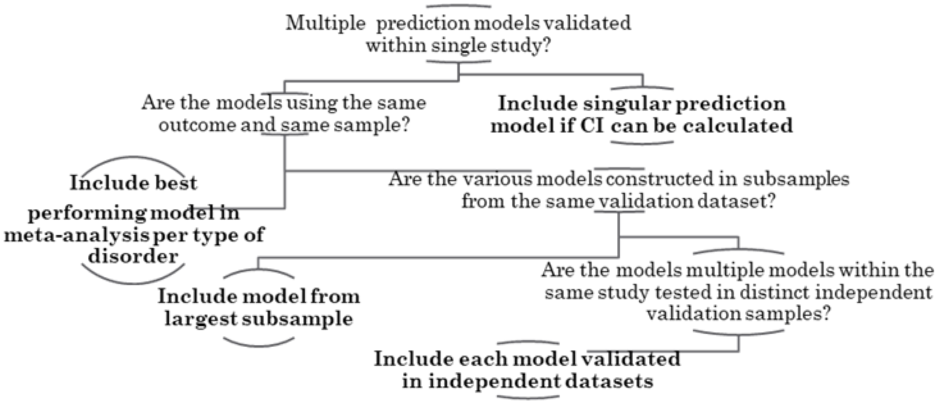

Caption: Decision-tree for including models into meta-analysis in case of multiple reported models per study.

6.3: PROBAST domain-specific and overall evaluation included studies

Title: PROBAST domain-specific and overall evaluation of included studies

Study	1. Participant		2. Predictors		3. Outcome		4. Analysis	Overall	
	RoB	App.	RoB	App.	RoB	App.	RoB	RoB	App.
Ashar.2021 (28)	Low	High	Low	High	Low	Low	High	High	High
Athreya.2019 (29)	Low	High	Low	High	Low	Low	High	High	High
Arthreya.2021 (30)	Low	Low	Unclear	Low	Low	Low	High	High	Low
Bone.2021 (31)	Low	Low	Low	Low	Low	High	Unclear	Unclear	High
Cattaneo.2016 (32)	Low	High	Low	High	Low	Low	High	High	High
Chekroud.2016 (33)	Low	High	Low	Low	Low	Low	High	High	High
Fabbri.2020 (34)	Low	Low	High	High	High	Low	High	High	High
Fazel.2017 (35)	Low	Low	Low	Low	Low	Low	High	High	Low
Fazel.2019 (36)	Low	Low	Low	Low	Low	Low	High	High	Low
Fiedorowicz.2021 (37)	Low	Low	Low	Low	Low	Low	Unclear	Unclear	Low
Furukawa.2019 (38)	Low	Low	Low	Low	Low	Low	Low	Low	Low
Hayes.2021 (39)	Low	Low	Low	Low	High	High	Unclear	High	High
Jha.2019a (40)	High	High	Low	Low	Low	Low	High	High	High
Jha.2019b (41)	High	High	Low	Low	Low	Low	High	High	High
Kambeitz-Ilankovic.2021 (42)	Low	High	Low	High	High	Low	High	High	High
Kautzky.2019 (43)	High	Low	High	Low	Unclear	Low	High	High	Low
Klein.2018 (44)	Low	High	Low	Low	Low	Low	High	High	High
Leighton.2019 (45)	Low	Low	Low	Low	Low	Low	High	High	Low
Leighton.2021 (46)	Low	Low	Low	High	Low	Low	High	High	High
Nie.2018 (47)	High	High	Unclear	Unclear	Low	Low	High	High	High
Nunez.2021 (48)	Low	High	Low	Low	Low	Low	High	High	High
Oritz.2020 (49)	High	High	Low	Low	High	Low	High	High	High
Perlis.2010 (50)	Low	Low	Low	Low	Low	Low	High	High	Low
Perry.2021 (51)	Low	Low	Low	Low	Low	Low	High	High	Low
Puntis.2021 (52)	Low	Low	Low	Low	Low	Low	Low	Low	Low
Soldatos.2022 (53)	Low	High	Low	Low	Low	Low	High	High	High
Taliaz.2021 (54)	Low	Low	Low	High	High	Low	High	High	High
Wang.2014 (55)	High	Low	High	Low	Low	Low	Low	High	Low

RoB= Risk of Bias, App.=Applicability

Caption: Overall risk of bias and applicability concerns for the majority of the studies was high.
