# Supplementary material for: Externally validated clinical prediction models for estimating treatment outcomes for patients with a mood, anxiety or psychotic disorder: systematic review and meta-analysis
Source: BJPsych Open. 2024 Dec 5;10(6):e221. doi: 10.1192/bjo.2024.789 (PMC11698186; doi:10.1192/bjo.2024.789)
Supplement: Burghoorn et al. supplementary material 6 — Burghoorn et al. supplementary material [file S2056472424007890sup006.docx]

Supplement 7 – Additional supplements

7.1 - Codebook

| ***Type*** | **Category** | **Study1 [name variable 1]; [name variable 2]** |
| --- | --- | --- |
| *Biological* | Age | Perlis.2010 [age at study entry (per 10 years)]; [age at onset (per 10 years)] Puntis.2021 [age]  Leighton.2021 [age t study entry] Wang.2014 [age]  Bone.2021 [Leeds Risk Index score (age)] Fazel.2017 [age (per 10 years)] Taliaz.2021 [age]  Furukawa.2019 [age] Nunez.2021 [age at interview] Fazel.2019 [age (per 10 years)]  Fiedorowicz.2021 [age]; [age of mood disorder onset]  Perry.2021 [age] |
| *Biological* | BMI | Hayes.2021 [sex by BMI group interaction]; [BMI group] Perry.2021 [BMI] |
| *Biological* | Ethnicity | Perlis.2010 [Hispanic vs. non-Hispanic] Chekroud.2016 [black or African American]; [White] Puntis.2016 [ethnicity]  Wang.2014 [white] Leighton.2019 [white] Hayes.2021 [BAME]  Perry.2021 [ethnicity] |
| *Biological* | Sex | Perlis.2010 [male sex] Puntis.2021 [gender] Leighton.2021 [male sex] Wang.2014 [female sex] Fazel.2017 [male sex] Hayes.2021 [sex] Nunez.2021 [sex]  Fazel.2019 [sex] |
| *Biological* | Genes | Fabbri.2020 [polygenetic risk score]  Taliaz.2021 [rs19291388]; [rs7201082]; [rs558025]  Athreya.2019 [TSPAN5]; [AHR]; [DEFB1]; [ERICH3] |
| *Biological* | Brain  biomarkers | Ashar.2021 [amygdala connectivity]  Kambeitz-Ilankovic.2021 [GM volume principal component scores] |
| *Biological* | GMH  (General medical history; comorbid somatic & physical  disorders) | Klein.2018 [chronic somatic illness]  Hayes.2021 [eGFR]; [baseline eGFR by ages interaction]; [hypothyroidism]; [migraine]; [high LDL cholesterol]; [hyperthyroidism]; [T2DM]  Nunez.2021 [physiological comorbidities]  Cattaneo.2016 [absolute number of MIF molecules]; [absolute number of IL-1β mRNA  molecules]  Perry.2021 [HDL concentration]; [triglyceride concentration] |
| *Table continues on next page* | | |

| ***Type*** | **Category** | **Study1 [name variable 1];[name variable 2]** |
| --- | --- | --- |
| *Clinical* | Depressed symptom (severity/frequency) | Kautzky.2019 [symptom severity]  Perlis.2010 [% days depressed, past year (per 10%)] Chekroud.2016 [QIDS]; [HAM-D]; [Have you been bothered by aches and pains in many different parts of your body?]; [Depressed mood most of the day, nearly everyday] Bone.2021 [PHQ-9]; [symptom severity]  Jha.2019a [QIDS-SR] Taliaz.2021 [HAM-D] Athreya.2019 [QIDS-C] Jha.2019b [QIDS-SR] Furukawa.2019 [PHQ9]; [BDI2] Nie.2018 [HAM-D] Nunez.2021 [HRS]; [IDSC];  Fazel.2019 [comorbid depression]  Arthreya.2021 [HDRS Depressed mood]; [HDRS guilt feelings and delusion]; [HDRS work and activities]; [HDRS psychic anxiety];  [strata change based on total HDRS scorel] |
| *Clinical* | (Comorbid) Diagnosis | Perlis.2010 [any comorbid axis I anxiety disorder]; [current alcohol abuse disorder]  Klein.2018 [Axis-I comorbidity] Kautzky.2019 [GAD]  Puntis [diagnosis (schizophrenia or non-schizophrenia)]; [substance abuse diagnosis]  Nie.2018 [any anxiety disorder]  Nunez.2021 [comorbid psychiatric disorders and family history  thereof] |
| *Clinical* | Anxiety symptom (severity/frequency) | Ashar.2021 [LSAS]  Perlis.2010 [% days anxious, past year (per 10%)]  Chekroud.2016 [Did any of the following make you fearful, anxious or nervous because you were afraid you'd have an anxiety attact in this situation (Standing in long lines, driving or riding in a car)]; [Did you have attacks of anxiety that caused you to avoid certain  situations or to change your behaviour or normal routine?] |
| *Clinical* | Global functioning and HRQOL | Leighton.2021 [GAF symptoms]; [GAF disability]; [PAS]; [PCT] Wang.2014 [SF-12 mental disability]; [SF-12 physical disability] Bone.2021 [Leeds Risk Index score (disability and functional impairment)]  Jha.2019b [activity impairment] Soldatos.2022os.2021 [GAF]; [PSP]  Nunez.2021 [improved psychological functioning]; [QLESQ];  [SFHS]; [WSAS] |
| *Clinical* | Insight scale-nervous or mental illness | Leighton.2021 [Insight scale-nervous or mental illness] |
| *Clinical* | PANSS Gx | Oritz.2020 [G9]  Leighton.2021 [G6] Leighton.2019 [G1]; [G6]; [G9]  Soldatos.2022os.2021[G12]; [G16] |
| Clinical | PANSS Nx | Oritz.2020 [N5]  Leighton.2021 [N4] Leighton.2019 [N3]; [N5]; [N4]  Soldatos.2022 [N6]; [N1]; [N2]; [N3]; [N4] |
| Clinical | PANSS Px | Oritz.2020 [P2] Leighton.2021 [P1]; [P2]; [P3]  Leighton.2019 [P4]  Soldatos.2022 [P1] |
|  | Table continues on next page | |

| *Type* | Category | Study1 [name variable 1];[name variable 2] |
| --- | --- | --- |
| *Clinical* | Suicidal risk/self-harm | Fazel.2017 [Previous self-harm] Hayes.2021 [self-harm]  Fazel.2019 [previous self-harm] |
| *Clinical* | PHX (psychiatric history; care consumption/previous episodes) | Kautzky.2019 [duration MDE >3 months]; [number of MDE]; [in- or outpatient]  Perlis.2010 [mood rapid cycling past year] Chekroud.2016 [number of previous MDE]  Puntis.2021 [number of days under EIP care] ; [number of previous admissions to a psychiatric hospital]  Klein.2018 [number of depressive episodes (2, 3 or 4, more than 5)]; [severity last episode]; [treatment PCT]  Leighton.2021 [DUP (days)]  Wang.2014 [MDD last year]; [2 or 3+ MDD]; [Lifetime GAD]; [Avoidant personality disorder]; Depressive symptoms in past MDE (difficulties in concentration, wanted to eat more, felt guilty)] Bone.2021 [Leeds Risk Index score (initial outcome expectancy)] Fazel.2017 [inpatient at time episode]; [length stay in days] Soldatos.2022 [DUP]  Furukawa.2019 [duration of episode] Nie.2018 [recurrent depression]  Nunez.2021 [history of comorbid psychiatric disorders] Fiedorowicz.2021 [maximum PSR score previous episode]; [number of weeks with threshold depression previous episode]; number of weeks mania/hypomania previous episode]; [remission variables]; [number of recurrences (1, 2 or more)]; [inclusion  hypomanic/manic symptoms in past episode(s)] |
| *Clinical* | MHX (medication history) | Chekroud.2016 [Ever taken sertraline]  Wang.2014 [Depressive symptoms in past MDE (ever took medication for low mood)]  Fazel.2017 [recent antipsychotic treatment]; [recent antidepressant treatment]; [treatment dependence] Hayes.2021 [SSRI exposure]  Furukawa.2019 [FIBSER]  Nunez.2021 [medication decision week 2]; [USQ (health care usage)]  Fazel.2019 [recent treatment (antipsychotic, antidepressant)] Perry.2021 [prescription of a metabolically active antipsychotic  drug] |
| *Psychosocial* | Family history | Fazel.2019 [parental psychiatric hospitalization]  Fiedorowicz.2021 [family history of mania] |
| *Psychosocial* | Alcohol use/substance abuse | Fazel.2017 [Previous alcohol use]  Fazel.2019 [previous alcohol use] |
| *Psychosocial* | Childhood adverse events | Klein.2018 [childhood adverse events] |
|  | Table continues on next page | |

| ***Type*** | **Category** | **Study1 [name variable 1];[name variable 2]** |
| --- | --- | --- |
| *Psychosocial* | Education | Chekroud.2016 [years]  Fazel.2017 [Education level (upper secondary, post-secondary) Taliaz.2021[years]  Leighton.2019 [education atainment]; [EET] Furukawa.2019 [years]  Nunez.2021 [years]  Fazel.2019 [ education level (upper secondary, post secondary)] |
| *Psychosocial* | Employment status | Chekroud.2016 [currently employed]  Bone.2021 [Leeds Risk Index score (employment)] Fazel.2017 [benefit recipient]  Taliaz.2021 [Status (Full-time employement, unemployment and not looking for a job)]  Nunez.2021 [employment status (paid or not-paid)  Fazel.2019 [benefit recipient] |
| *Psychosocial* | Geographical deprivation index LSOA | Puntis.2021 [Geographical area depreviation index (income deprivation, education skill and training development, health deprivation and disability, barriers to housing and services,  environmental deprivation, employment deprivation)] |
| *Psychosocial* | Income | Perlis.2010 [household income <$50k]  Fazel.2017 [personal income (2nd to 10th deceile)]  Nunez.2021 [income] |
| *Psychosocial* | Household composition | Leighton.2019 [Living with spouse & children]; [private accomodation]; [rented accomodation]  Nunez.2021 [household] |
| *Psychosocial* | Drug use | Leighton.2021 [past drug use] Fazel.2017 [Previous drug use] Fazel.2019 [previous drug use] Hayes.2021 [smoking status]  Perry.2021 [current smoking status] |
| *Psychosocial* | Past violent crime | Fazel.2017 [Previous violent crime]  Fazel.2019 [previous violent crime] |
| *Psychosocial* | Relationship status (Married/common-law or divorced/separated/single;  in a relationship) | Wang.2014 [ Married/common-law, Divorced/separated/single] Taliaz.2021 [marital status]  Leighton.2019 [In a Relationship]  Nunez.2021 [relationship status] |
| *Psychosocial* | Family drug and alcohol use | Fazel.2017 [parental drug or alcohol use]  Fazel.2019 [parental drug or alcohol use] |
| *Psychosocial* | Family violent crime | Fazel.2017 [sibling violent crime]; [parental violent crime] |
| *Psychosocial* | Trauma events | Chekroud.2016 [Did reminders of a traumatic event make you shake, break out into sweat or having a racing heart?] ; [Have you ever witnessed a traumatic event such as rape, assault, someone dying in an accident, or any other extremely upsetting event?]; [Did you try to avoid activities, places, or people that reminded you of a traumatic event?]  Fazel.2019 [parental suicide] |
| *Psychosocial* | Care economic status | Nunez.2021 [medical leave]; [insurance (private or Medicare)] |

7.2 - R script

setwd("~/R/Desi")

#install packages install.packages("metafor") library(metafor) install.packages("readxl") library(readxl) help(metafor)

install.packages("metamisc") library(metamisc)

Meta_c<-read_xlsx("Meta extractie_22-03-2022.xlsx")

est1 <- ccalc(cstat = c.index, cstat.se = se.c.index, cstat.cilb = c.index.95CIl, cstat.ciub = c.index.95CIu, N = n, O = n.events, data = Meta_c)

#studienaam toevoegen en missings verwijderen est1$slab <- Meta_c$AuthorYear

est1$study <- Meta_c$Study est1$sample <- Meta_c$Sample est1$model <- Meta_c$Model est1$outcome <- Meta_c$Outcome

est1$outcomesympt <- Meta_c$Outcome_sympt est1$outcometype <- Meta_c$Outcome_type est1$include <- Meta_c$Include

est1$Discr <- Meta_c$Discrimination.measure

#Meta-analysis of the c-statistic (random effects)

fit <- valmeta(cstat=theta, cstat.se=theta.se, cstat.cilb=theta.cilb, cstat.ciub=theta.ciub, cstat.cilv=0.95, slab = slab, data=est1)

plot(fit) print(fit) fit

#Sensitivity analysis of exclusing Single Accuracy Cut-off sens_analysis <- est1[-c(3, 4, 5, 13, 14, 25, 26, 27), ]

fit1 <- valmeta(cstat=theta, cstat.se=theta.se, cstat.cilb=theta.cilb, cstat.ciub=theta.ciub, cstat.cilv=0.95, slab = slab, data=sens_analysis)

plot(fit1) print(fit1) fit1

#Get the logit transformed values from Metamisc. est2<-fit$data

#Meta-analysis of the c-statistic (random effects)

fit <- valmeta(cstat=theta, cstat.se=theta.se, cstat.cilb=theta.cilb, cstat.ciub=theta.ciub, cstat.cilv=0.95, slab = slab, data=est1)

#Replicate results with metafor package. #Make variables.

est2$yi<-est2$theta est2$vi_se<-est2$theta.se est2$vi<- (est2$theta.se)^2

#Run meta analysis.

res <- rma(yi, vi, data=est2) res

#predict(res, digits=4). Terug transformeren van model resultaten. predict(res, transf=transf.ilogit, digits=4)

#Funnel met metafor funnel(res, atransf=transf.ilogit)

funnel(res, atransf=transf.ilogit.int, xlab = "Discrimination")

#meta-regressie op basis van type disorder, depressie vs. other. est2$dep <- ifelse(est2$outcomesympt== "dep", 1,0)

res_tf0<- rma(yi, vi, mods = ~ dep, data=est2) res_tf0

predict(res_tf0, transf=transf.ilogit, digits=4)

#controle -- meta-regressie op basis van type disorder, smi ('other') vs. depressie est2$anx <- ifelse(est2$outcomesympt== "anx", 1,0)

est2$psy <- ifelse(est2$outcomesympt== "psy", 1,0)

est2$smi <- ifelse((est2$outcomesympt== "psy" | est2$outcomesympt== "bp" | est2$outcomesympt== "smi"), 1,0)

res_tf<- rma(yi, vi, mods = ~ smi, data=est2) res_tf

predict(res_tf, transf=transf.ilogit, digits=4)

#meta-regressie op basis van aantal predictoren est2$prednum <- Meta_c$Pred_numb

res2 <- rma(yi,vi,mods =prednum, data=est2) res2

predict(res2, transf = transf.ilogit, digits=4)

#meta-regressie op basis van type factoren (biopsysocclin. vs. other) table(Meta_c$Pred_BioClinSoc)

est2$BioClinSoc <- Meta_c$Pred_BioClinSoc

res_tf1 <- rma(yi,vi,mod=est2$BioClinSoc, data=est2) res_tf1

#meta-regressie op basis van type factoren (clinical model vs. other) library(dplyr)

Meta_c$Pred_Clin_Only<-

if_else(Meta_c$Pred_bio==0 & Meta_c$Pred_soc==0 & Meta_c$Pred_clin==1, 1, 0)

)

table(Meta_c$Pred_Clin_Only) est2$Pred_Clin_Only <- Meta_c$Pred_Clin_Only

res_tf5 <- rma(yi, vi, mod=~Pred_Clin_Only, data=est2) res_tf5

#meta-regressie op basis van type factoren (Biological model vs. other) Meta_c$Pred_Bio_Only<-

if_else(Meta_c$Pred_bio==1 & Meta_c$Pred_soc==0 & Meta_c$Pred_clin==0, 1, 0)

)

table(Meta_c$Pred_Bio_Only) est2$Pred_Bio_Only <- Meta_c$Pred_Bio_Only

res_tf5 <- rma(yi, vi, mod=est2$Pred_Bio_Only, data=est2) res_tf5

#checking models using biological and psychosocial variables only Meta_c$Pred_Bio_Soc<-

if_else(Meta_c$Pred_bio==1 & Meta_c$Pred_soc==1 & Meta_c$Pred_clin==0, 1, 0)

)

table(Meta_c$Pred_Bio_Soc) #zero models

#checking models using both clinical and psychosocial variables only Meta_c$Pred_Clin_Soc<-

if_else(Meta_c$Pred_bio==0 & Meta_c$Pred_soc==1 & Meta_c$Pred_clin==1, 1, 0)

)

table(Meta_c$Pred_Clin_Soc) #zero models

7.3 Endpoint definition per validation dataset

| **ID** | **Instrument** | **Endpoint_def** |
| --- | --- | --- |
| **Ashar.2021** | LSAS | Pre-to-post treatment change in LSAS score with traditional cut- offs for mild, moderate and severe at 30, 50 and 90. |
| **Oritz.2020** | PANNS | Inpatients: symptom change from baseline. Outpatients: cross- sectional recommendations based on persistence of selected symptoms (P1-delusions, G9-unusual thought content, P3- hallucinatory behavior, G5-mannerisms/posturing, N1-blunted effect, N4-social withdrawal, N6-lack of spontaneity) rating on  moderate to severe levels |
| **Fabbri.2020** | MADRS, QIDS-C16 | GENDEP: MADRS Response: <22 and >=50% reduction compared to onset of current MDD episode, STAR*D: IDS-C16 Response:<13  and score decrease of >=50% at level 1 exit |
| **Kautzky.2019** | MADRS | Response: MADRS <=21 score at inclusion, as well as a decline from baseline to current MADRS of >= 50% OR TRD: failed treatment response after two or more consecutive AD or combination or augmentation therapy of adequate duration (>=4  weeks) and dosage were administered |
| **Perlis.2010** | Clinical Monitoring  Form | Reported percentage of missed milligrams per study drug in the past week. Poor adherence defined as missing at least 25% of  total doses of any 1 medication. |
| **Chekroud.2016** | QIDS-SR16 | Total score <=5 |
| **Puntis.2021** | NA | Yes/No admission to an inpatient psychiatric unit |
| **Klein.2018** | SCID-I | Yes/No |
| **Leighton.2021** | PANNS | Score <=3 points in PANNS items P1 Delusions, P2 Conceptual Disorganisation, P3 Hallucinatory Behaviour, N1 Blunted Affect, N4 Apathetic Social Withdrawal, N6 Lack of Spontaneity, G9  Unusual Thought Content. Present for at least 6 months |
| **Wang.2014** | AUDADIS-IV | Yes/No |
| **Bone.2021.dep** | PHQ-9 | Below the relevant cutoff score for each outcome measure, and to have improved by a magnitude greater or equal to the reliable  change index relative to the baseline measure. |
| **Bone.2021.anx** | GAD-7 | Below the relevant cutoff score for each outcome measure, and to have improved by a magnitude greater or equal to the reliable  change index relative to the baseline measure. |
| **Fazel.2017** | NA | Yes/No |
| **Ja.2019a.Remission** | QIDS-C | Total score <= 5 |
| **Jha.2019a.nomeaningfulbenefit** | QIDS-C | <30% reduction from baseline |
| **Kambeitz-Ilankovic.2021** | GAF-scale for DSM-IV | Lower functioning: total score <45, higher functioning: total score>=45 |
| **Taliaz.2021. classic** | QIDS-C | Exponential fit for the personal measurements of QIDS (i.e.,  “exponential antidepressant response”). Below median=no  response, above median=response |
| **Taliaz.2021.exponential** | QIDS-C | 50% QIDS score reduction (i.e., “classic antidepressant response”):  (last day score - initial score participant same treatment)/initial  score. Responder: >=0.5. Non responder: <0.5. |
| **Athreya.2019** | QIDS-C, HDRS | Remission: total score HDRS <= 7, total score QIDS-C<=5. Response: >= 50% decline in scores without remission from  baseline to 4 or 8 weeks |
| Table continues on next page | | |

| ***(continued) Table supplement 8.3: Endpoint definition per validation dataset*** | | |
| --- | --- | --- |
| **ID** | **Instrument** | **Endpoint_def** |
| **Leighton.2019** | EET, PANNS | EET: employment education or training status one year. PANNS point remission: meeting Andreasen PANNS criteria at month 12. PANNS period remission: meeting Andreasen PANSS criteria at both month 6 and month 12. Andereasen defined remission as  <=3 in PANNS items P1 delusions, P2 Conceptual Disorganisation, P3 Hallucinatory Behaviour, N1 Blunted Affect, N4 Apathetic Social Withdrawal, N6 Lack of Spontaneity and G9 Unusual  Thought Content, present for a period of at least 6 months. |
| **Jha.2019b.Remission** | QIDS-SR | Total score <=5 |
| **Ja.2019b.nomeaningfullbenefit** | QIDS-SR | <30% decrease from baseline |
| **Soldatos.2022** | PANNS | Score <=3 in PANNS items P1 delusions, P2 Conceptual Disorganisation, P3 Hallucinatory Behaviour, N1 Blunted Affect, N4 Apathetic Social Withdrawal, N6 Lack of Spontaneity and G9 Unusual Thought Content, present for a period of at least 6  months. Due to study time, waived 6-moth criterion |
| **Hayes.2021** | - | Risk score predicting eGFR decline |
| **Hayes.2021.eGFR** | - | Stage 3a kidney disease or higher |
| **Furukawa.2019** | PHQ-9 | Remission risk status (low vs. high) |
| **Nie.2018.Remission** | HAM-D17 | Total score <=7, Response: 50% baseline reduction in HAM-D score. |
| **Nie.2018.Response** | HAM-D17 | Response: 50% baseline reduction in HAM-D score. |
| **Nunez.2021.**  **Remission** | QIDS-SR | Total score <=5 |
| **Nunez.2021. Response** | QIDS-SR | >= 50% decline in scores without remission from baseline upto week 8. |
| **Fazel.2019** | - | Yes/No |
| **Fiedorowicz.2021** | SADS | Yes/No (hypomania, mania, depressive episodes) |
| **Arthreya.2021.SSRI/SNRI/COMED. Remission** | HDRS | Remission ≤ 7 |
| **Arthreya.2021.SSRI/SNRI/COMED.**  **Response** | HDRS | ≥50% reduction either 4 or 8 weeks of treatment. |
| **Cattaneo.2016** | MADRS | ≥50% reduction |
| **Perry.2021** | - | at least three of the following : ethnicity-specific waist cir- cumference of at least 94 cm in males and at least 80 cm in females for white people, at least 90 cm in males and at least 80 cm in females for other ethnic groups, or body-mass index (BMI) greater than 29·9 kg/m2; triglyceride concentrations at least 1·70 mmol/L; HDL concentration less than 1·03 mmol/L in males or less than 1·29 mmol/L in females; systolic blood pressure greater than  130 mm Hg; or fasting plasma glucose greater than 5·60 mmol/L. |

7.4 Characteristics included models in meta-analysis

| ***Table supplement 8.4: Characteristics included models in meta-analysis.*** | | | | | | | |
| --- | --- | --- | --- | --- | --- | --- | --- |
| *Study* | Disor der | Sample & subcohort | Model | Mode l type | CI 95%* | Discr. Measur  e | Outco me |
| *Fabbri.2020* | Mood (DEP) | Star*D | Rare and common  variants | ML | 0.600 (0.540-  0.650) | c-stat | TR |
|  |  | GENDEP | Rare variants |  | 0.720 (0.580-  0.860) |  |  |
| *Chekroud.2016* | Mood (DEP) | COMED | Esc + placebo | ML | 0.596 (0.513-  0.675) | ASC | RE |
|  |  |  | Esc+Bup |  | 0.597 (0.428-  0.600) |  |  |
|  |  |  | Vem+Mir |  | 0.514 (0.509-  0.681) |  |  |
| *Puntis.2021* | Psy | NHS trust; West London | - | Lo | 0.700 (0.660-  0.750) | c-stat | HA |
| *Leighton.2021* | Psy | Outlook study | - | Lo | 0.730 (0.710-  0.750) | c-stat | RE |
| *Wang.2014* | Mood  (DEP) | NESARC; South and  West Region | - | Lo | 0.720 (0.687-  0.752) | c-stat | REL |
| *Bone.2021* | Mood (DEP) | NHS Trust; Cumbria, Northumberland, Tyne  and Wear | Oracle2 | Lo | 0.614 (0.578-  0.650) | c-stat | TRE |
|  |  | NHS Trust; Whittington, Barnet, Enfield, Haringey, Pennine and  Humber. | Extreme gradient | ML | 0.623 (0.578-  0.650) |  |  |
| *Fazel.2017* | SMI | Swedish national cohort  with crime register | - | Lo | 0.890 (0.850-  0.930) | c-stat | CC |
| *Jha.2019a* | Mood (DEP) | SAMS | - | Lo | 0.840 (0.778-  0.901) | c-stat | NMB |
| *KambeitzIlank*  *ovic.2021* | Psy | CRIS | - | ML | 0.694 (0.459-  0.929) | AUC | GF |
| *Taliaz.2021* | Mood  (DEP) | PGRN-AMPS | - | ML | 0.613 (0.504-  0.722) | AUC | TRE |
| *Leighton.2019* | Psy | NHS Trust, Glasgow and Edinburgh | - | ML | 0.876 (0.864-  0.887) | c-stat | EET |
|  |  |  |  | ML | 0.652 (0.635-  0.670) |  | RE |
| *Jha.2019b* | Mood (DEP) | COMED | - | Lo | 0.822 (0.770-  0.874) | c-stat | NMB |
| *Soldatos.2022* | Psy | Copenhagen Cohort (  PECANSI & PECANSII) | - | ML | 0.6774 (0.564-  0.790) | c-stat | RE |
| *Hayes.2021* | Mood (BP) | CRPD Gold | Elastic net | Lo | 0.888 (0.864-  0.912) | c-stat | AE |
| *Furukawa.201*  *9* | Mood  (DEP) | SUNJ | Week 1 | Lo | 0.730 (0.700-  0.770) | c-stat | RE |
| *Nie.2018* | Mood (DEP) | RIS-INT-93 | PLR | ML | 0.780 (0.697-  0.863) | c-stat | TRE |
| *Nunez.2021* | Mood  (DEP) | CAN-BIND-1 | Random forests | ML | 0.829 (0.758-  0.899) | c-stat | RE |
| *Table continues on next page* | | | | | | | |

| ***(continued) Table supplement 8.4: Characteristics included models in meta-analysis.*** | | | | | | | |
| --- | --- | --- | --- | --- | --- | --- | --- |
| *Fazel.2019* | SMI | Swedish national cohort with total population  register | - | Lo | 0.710 (0.660-  0.750) | c-stat | SU |
| *Fiedorowicz.20 21* | Mood (BP) | CDS | 1year anypolarity | ML | 0.779 (0.765-  0.793) | c-stat | REL |
| *Arthreya.2021* | Mood (DEP) | ISPC and Eli.Lilly.and.Co. | Par+Flu+Ser,  path SSRI.A2.B2 | ML | 0.620 (0.481-  0.759) | ASC | TRE |
|  |  | Eli Lilly and Co | Dul, path SSRI.A2.B2 | ML | 0.620 (0.457-  0.783) |  |  |
|  |  | MARS | SSRI.A2.B2 | ML | 0.600 (0.462-  0.738) |  |  |
| *Perry.2021* | Psy | SLaM EIS | Full model | Lo | 0.750 (0.690-  0.800) | c-stat | AE |
| ***Note: AE= adverse effects medication, ASC= accuracy at single cutoff, AUC= area under the curve, BP= Bipolar Disorder, Bup=Buporpion, CC=crime committance, C-stat= c-statistic, DEP= depressive disorder, Dul= Duloxetine, EET=Education Employment Training status, Esc= Escitalopram, Flu=Fluoxetine, GF=Global functioning, HA=Hospital admission, Li=linear regression, Lo=Logistic regression, ML= machine learning, Mir=Mirtazapine, NMB=no meaningful benefit, , Psy=Psychosis, SMI= severe mental disorders (mixed sample), SSRI-serotonin and noradrenaline reuptake inhibitor, SU=Suicide, TR= treatment resistance, TRE=treatment response RE=remission, REL=relapse, R2= R square, Ven=Venlafaxine***  ****Confidence interval (CI) rounded on three decimals*** | | | | | | | |

7.5 - Characteristics included models in meta-analysis

| ID | Learning | | Type | Selection criteria predictors | Enter method predictor into model | Handling missing data |
| --- | --- | --- | --- | --- | --- | --- |
|  | Stat | ML |  |  |  |  |
| Ashar.2021 | X |  | Li | None | Full | LOCV |
| Oritz.2020 | X |  | Log | p-value | Forward | UNK |
| Fabbri.2020 |  | X | GBM | LFDR | Full | Removal, FreeBayes |
| Kautzky.2019 | X |  | Log | p-value | Full | CCA |
| Perlis.2010 | X |  | Log | p-value | Backward | CCA |
| Chekroud.2016 |  | X | GBM | ABW | Full | CCA |
| Puntis.2021 | X |  | Log | cf, Lit | Full | Imp (UNK) |
| Klein.2018 | X |  | Log | p-value | Full | CE |
| Leighton.2021 | X |  | Log | Ep, Lit | Full | CE |
| Wang.2014 | X |  | Log | p-value | Forward, backward | Hot-deck |
| Bone.2021 | X | X | Log, Bay, EN, XGBoost, SVM | ABW, p-value | Backward, various forms | CCA |
| Fazel.2017 | X |  | Log | p-value | Full | Removal, CE |
| Jha.2019a | X |  | Log | UNK | Full | CCA |
| Kambeitz- Ilankovic.2021 |  | X | SVM | Grid | Full | CCA |
| Taliaz.2021 |  | X | SVM, XGBoost, RF, AdaBoost | ABW | Full | Removal, CE |
| Athreya.2019 |  | X | RF | PDF | Full | Imp (UNK) |
| Leighton.2019 |  | X | EN | ABW | Full | Imp |
| Jha.2019b | X |  | Log | UNK | Full | k-nearest |
| Soldatos.2021 |  | X | EN | ABW | Full | MICE |
| Hayes.2021 | X |  | Log | ABW, Lit | Full | None |
| Furukawa.2018 | X |  | Log | p-value | Backward | MI |
| Nie.2018 |  | X | RF, GBM, XGBoost, PLR, EN | ABW | Full | UNK |
| Nunez.2021 |  | X | RF, GBDT, XGBoost, PLR, EN | ABW | Full | Enter central measure, hot-coding |
| Fazel.2019 | X |  | Log | p-value | Forward, backward | Removal, MI |
| Fiedorowicz.2021 |  | X | BCT | None | Full | UNK |
| Arthreya.2021 |  | X | HM | NIR | Full | UNK |
| Cattaneo.2016 | X |  | LDF | None | Full | UNK |
| Perry.2021 | X |  | Log | None | Full | CE |
| ABW=Absolute beta weights, AdaBoost=Adaptive Boosting, Bay=Bayesian updating algorithm, BCT=Boosted classification trees, CCA=Complete case analysis, CE=Chained equations, cf=clinical feasibility, EN=Elastic Net, Ep= expert panel, Full= All at once, GBDT = Gradient boosting decision trees, GBM=gradient boosting, Grid=Grid search hyperparameters, HM=Hidden Markov model, Imp=Imputation, RF=Random forests, k-nearest = k nearest neighbor imputation, LOCV=Last observation carried forward, ML=Machine learning, MI=Multiple imputation (not specified), MICE = multivariate imputation chained equations, NIR=Null information rate, NN=neural networks, PDF=Probabilty density functions (Clustering), PLR=Penalized logistic regression, Stat=statistical learning, SVM=support vector machine, LDF=Linear discriminant function, LFDR=Local false discovery rate, Li=linear regression, Lit=Literature, log=logistic regression, UA=unadjusted associations with outcome variable significant, UNK=Unknown, XGBoost=eXtreme Gradient Boosting, | | | | | | |

## 7.6 – Comparison between Furukawa et al. 2019 & Puntis et al. 2021

| Table S7.6: Comparison between the two studies scoring of low concern on both PROBAST applicability and risk of bias | | |
| --- | --- | --- |
|  | **Furukawa et al. 2019**  **(PMID: 30430961)** | **Puntis et al. 2021**  **(PMID: 33431803)** |
| Source validation dataset | Clinical trials (pragmatic close to clinical practice) | Observation cohort |
| Participants | Mood disorder diagnosis  *Adults* | Psychotic disorder diagnosis  *Adults* & adolescents |
| Treatment outcome | *Clinical*: remission  Endpoint: week 1, week 3 | *Clinical*: hospital readmission one year after discharge  Endpoint: 12 months |
| Predictors | *Clinical*: PHQ-9, BDI-II, FIBSER, length of episode  *Psychosocial*: Education  *Biological: Age* | *Clinical*: Schizophrenia diagnosis, duration of EIP, Number of previous admissions, substance misuse diagnosis  *Psychosocial*: LSOA  *Biological:* Gender, Ethnicity, *Age* |
| Performance measures | Discrimination: AUC  Calibration: HLT  Other: PPV, NPV, Spec, Sens | Discrimination: C-stat  Calibration: slope  Other: Brier |
| Modelling methods | *Machine learning: logistic regression* | *Machine learning: logistic regression* |
| Missing data handling | *Chained equations* | *Chained equations* |
| Type of external validation | Temporal validation | Geographic validation |
| Note: AUC=area under the curve, BDI-II=Back Depression Inventory second edition, Brier= brier score, C-stat=C-statistic, EIP= early intervention in psychosis services, FIBSER=frequency intensity and burden of side effects rating, HLT=hosmer-lemeshow test, LSOA=lower super output area – measure of local area deprivation, PHQ-9=patient health questionnaire-9, PPV=positive predictive value, Spec=specificity, Sens=sensitivity, SMI=severe mental illness.  *Cursive font* indicates a shared characteristic between the two studies | | |

## 7.7 – Comparison between highest quality studies

| **Table S7.7: evaluation of discrimination performance of included studies in their development datasets** | | | | |
| --- | --- | --- | --- | --- |
| **ID** | **Internal validation discrimination*** | | **Measure** | **Updating model** |
|  | **Lowest**** | **Highest** |  |  |
| Ashar.2021 | - | Excellent | AUC |  |
| Athreya.2019 | Acceptable | Excellent | AUC |  |
| Arthreya.2021 | Poor/Acceptable | Acceptable/Excellent | AUC | x |
| Bone.2021.anx | Poor | Excellent | AUC | x |
| Bone.2021.dep | Poor | Excellent | AUC | x |
| Cattaneo.2016 | N/A, no discrimination measure reported, only calibration | | | |
| Chekroud.2016 | - | Acceptable | AUC |  |
| Fabbri.2020 | Poor | Acceptable | AUC | x |
| Fazel.2017 | - | Excellent | C-stat |  |
| Fazel.2019 | - | Acceptable | C-stat |  |
| Fiedorowicz.2021 | - | Excellent | AUC |  |
| Furukawa.2019 | Poor | Excellent | AUC | x |
| Hayes.2021 | Excellent | Excellent | AUC |  |
| Jha.2019a | Acceptable | Acceptable | AUC |  |
| Jha.2019b | Excellent | Excellent | AUC |  |
| Kambeitz-Ilankovic.2021 | - | Poor | BAC |  |
| Kautzky.2019 | - | Excellent | ACC |  |
| Klein.2018 | - | Poor | C-stat |  |
| Leighton.2019 | No discrimination measures reported, but raw data available for replication analysis | | | |
| Leighton.2021 | - | Acceptable | C-stat |  |
| Nie.2018 | Poor | Poor | ACC |  |
| Nunez.2021 | Acceptable | Excellent | AUC |  |
| Oritz.2020 | Acceptable | Excellent | AUC |  |
| Perlis.2010 | - | Poor | AUC |  |
| Perry.2021 | Acceptable | Excellent | C-stat |  |
| Puntis.2021 | - | Acceptable | C-stat |  |
| Soldatos.2021 | Acceptable | Acceptable | AUC |  |
| Taliaz.2021 | Poor | Excellent | BAC |  |
| Wang.2014 | - | Acceptable | C-stat |  |
| Note: ACC=Accuracy, AUC=Area Under the curve, C-stat=C-statistic, BAC=Balanced Accuracy Curve,  *Evaluation based on ranges as stated in Hosmer & Lemeshow (2000); No discrimination= ≤0.5, modest= >0.5-<0.7, Acceptable=≥0.7 - <0.8, Excellent=≥0.8 - <0.9. **Not applicable if only one discrimination measure was reported. | | | | |

Hosmer, D.W. & Lemeshow, S. (2000). Applied Logistic Regression (2ed), 160-164. Wiley Series in probability and statistics. ISBN:0-471-35632. Accessed via: https://ftp.idu.ac.id/wp-content/uploads/ebook/ip/REGRESI%20LOGISTIK/epdf.pub_applied-logistic-regression-wiley-series-in-probab.
