# Supplementary material for: Externally validated clinical prediction models for estimating treatment outcomes for patients with a mood, anxiety or psychotic disorder: systematic review and meta-analysis
Source: BJPsych Open. 2024 Dec 5;10(6):e221. doi: 10.1192/bjo.2024.789 (PMC11698186; doi:10.1192/bjo.2024.789)
Supplement: Burghoorn et al. supplementary material 7 — Burghoorn et al. supplementary material [file S2056472424007890sup007.pdf]

Supplement 2 – search strings

Pubmed

("Mood Disorders"[MeSH] OR "Depression"[MeSH] OR "mood disorder*"[tiab] OR "affective disorder*"[tiab] OR "depressive disorder*"[tiab] OR “depression*”[tiab] OR "Bipolar and Related Disorders"[MeSH] OR "bipolar"[tiab] OR "manic"[tiab] OR "Anxiety Disorders"[MeSH] OR "anxiet*"[tiab] OR "Schizophrenia Spectrum and Other Psychotic Disorders"[MeSH] OR "psychoti*"[tiab] OR "psychos*"[tiab] OR “schizophren*"[tiab])
AND ((“external*“[tiab] *AND* (“validat*”[tiab] *OR* "replicat*"[tiab])) OR ("independent*"[tiab] *AND* "replicat*"[tiab]) OR "independent validat*"[tiab] OR "independently validat*"[tiab] OR (("Clinical Decision Rules"[MeSH] OR “clinical decision rule*”[tiab]) *AND* "replic*"[tiab]))
AND ("prognos*"[tiab] OR "predict*"[tiab] OR “classif*”[tiab] OR "decision*"[tiab])
AND ("treatment*"[tiab] OR “clinical*"[tiab] OR “patient*”[tiab] OR “mental health care*”[tiab] OR “psychiatric care*”[tiab] OR "Mental Health Services"[MeSH] OR “mental health service*”[tiab])
NOT ("Systematic Review"[Publication Type] OR "Review"[Publication Type] OR "Meta-analysis"[Publication Type] OR "review"[ti])

Embase

(‘Mood Disorder’/exp OR ‘mood disorder*’:ab,ti OR (‘depression*’ OR ‘affective disorder*’ OR ‘depressive disorder*’):ab,ti OR (‘bipolar’ OR ‘manic’):ab,ti OR ‘anxiety disorder’/exp OR ‘anxiet*’:ab,ti OR 'schizophrenia spectrum disorder'/exp OR ‘schizophren*’:ab,ti OR ‘psychosis’/exp OR (‘psychosis’ OR ‘psychoses’ OR ‘psychoti*’):ab,ti)
((‘external*’:ti,ab *AND* (‘validat*’:ti,ab *OR* ‘replicat*’:ti,ab)) OR (‘independent*’:ti,ab *AND* ‘replicat*’:ti,ab) OR ‘independent validat*’:ti,ab OR ‘independently validat*’:ti,ab OR<br><br>(('clinical decision rule'/exp *OR* ‘clinical decision rule*’:ti,ab) *AND* ‘replic*’:ti,ab))
(‘prognos*’ OR ‘predict*’ OR ‘classif*’ OR ‘decision*’):ti,ab
((‘treatment*’ OR ‘clinical*’ OR ‘patient*’ OR ‘mental health care*’ OR ‘psychiatric care*’):ti,ab OR 'mental health care’/exp OR ‘mental health service*’:ti,ab)
NOT ('systematic review'/exp OR 'review'/exp OR 'meta analysis'/exp OR ‘review’:ti)

PsychINFO search string can be found on the next page.

PsycINFO

(DE "Affective Disorders" OR DE "Disruptive Mood Dysregulation Disorder" OR DE "Major Depression" OR DE "Seasonal Affective Disorder" OR (TI "mood disorder*" OR AB "mood disorder*") OR (TI "affective disorder*" OR AB "affective disorder*")<br><br>OR (DE “Major Depression” OR DE “Dysthymic Disorder” OR DE “Endogenous Depression” OR DE “Late Life Depression” OR DE “Reactive Depression” OR DE “Recurrent Depression” OR DE “Treatment Resistant Depression”)<br><br>OR (TI "depressive disorder*" OR AB "depressive disorder*") OR (TI depression* OR AB depression*)<br><br>OR (DE "Bipolar Disorder" OR DE "Bipolar I Disorder" OR DE "Bipolar II Disorder" OR DE "Cyclothymic Disorder" OR DE "Mania") OR (TI bipolar OR AB bipolar) OR (TI manic OR AB manic)<br><br>OR (DE "Anxiety Disorders" OR DE "Castration Anxiety" OR DE "Generalized Anxiety Disorder" OR DE "Obsessive Compulsive Disorder" OR DE "Panic Attack" OR DE "Panic Disorder" OR DE "Phobias" OR DE "Separation Anxiety Disorder" OR DE "Trichotillomania")<br><br>OR (TI anxiet* OR AB anxiet*)<br><br>OR (DE "Acute Psychosis" OR DE "Acute Schizophrenia" OR DE "Affective Psychosis" OR DE "Alcoholic Psychosis" OR DE "Alcoholic Hallucinosis" OR DE "Capgras Syndrome" OR DE "Chronic Psychosis" OR DE "Experimental Psychosis" OR DE "Hallucinosis" OR DE "Alcoholic Hallucinosis" OR DE "Paranoia (Psychosis)" OR DE "Folie A Deux" OR DE "Reactive Psychosis" OR DE "Schizophrenia" OR DE "Acute Schizophrenia" OR DE "Catatonic Schizophrenia" OR DE "Paranoid Schizophrenia" OR DE "Process Schizophrenia" OR DE "Schizoaffective Disorder" OR DE "Schizophrenia (Disorganized Type)" OR DE "Schizophreniform Disorder" OR DE "Undifferentiated Schizophrenia" OR DE "Senile Psychosis" OR DE "Toxic Psychoses")
(((TI external* OR AB external*) AND ((TI validat* OR AB validat*) OR (TI replicat* OR AB replicat*))) OR ((TI independent* OR AB independent*) AND (TI replicat* OR AB replicat*)) OR ((TI "independent validat*" OR AB "independent validat*")) OR ((TI "independently validat*" OR AB "independently validat*")) OR (((TI "clinical decision rule*" OR AB "clinical decision rule*")) AND (TI replic* OR AB replic*)))
((TI prognos* OR AB prognos*) OR (TI predict* OR AB predict*) OR (TI classif* OR AB classif*) OR (TI decision* OR AB decision*))
((TI treatment* OR AB treatment*) OR (TI clinical* OR AB clinical*) OR (TI patient* OR AB patient*) OR (TI "mental health care*" OR AB "mental health care*") OR (TI "psychiatric care*" OR AB "psychiatric care*") OR<br><br>DE "Mental Health Services" OR DE "Community Mental Health Services" OR DE "Psychological First Aid"<br><br>OR (TI "mental health service*" OR AB "mental health service*"))
NOT<br>(PT "Systematic Review" OR PT “Literature Review” OR TI review)
